# Effects of Sevoflurane Anesthesia on Cerebral Lipid Metabolism in the Aged Brain of Marmosets and Mice

**DOI:** 10.3389/fnmol.2022.915570

**Published:** 2022-07-06

**Authors:** Haoli Mao, Jiao Zhu, Yanyong Cheng, Lingling Shi, Xiao Chen, Ren Zhou, Zhenyu Xue, Siyu Liu, Zilong Qiu, Hong Jiang

**Affiliations:** ^1^Department of Anesthesiology, Shanghai Ninth People’s Hospital, Shanghai Jiao Tong University School of Medicine, Shanghai, China; ^2^State Key Laboratory of Neuroscience, CAS Center for Excellence in Brain Science and Intelligence Technology, Shanghai Center for Brain Science and Brain-Inspired Intelligence Technology, Institute of Neuroscience, Chinese Academy of Sciences, Shanghai, China

**Keywords:** sevoflurane, general anesthesia, brain, lipid metabolism, marmosets, mice

## Abstract

**Objective:**

In the lipid-rich brain, lipids performed signaling processes associated with the control system of the cell cycle, stress, and inflammatory reactions, as well as maintained brain and cellular homeostasis. The effects of general anesthesia on brain impairment in the elderly were controversial and complex. The study sought to evaluate the effect of lipid metabolism in the brain of aged marmosets and mice under long-term exposure to sevoflurane.

**Methods:**

A total of 6 marmosets over 8-year-old and 10 mice aged 18 months were divided into the sevoflurane anesthesia and control groups, respectively. Marmosets in the sevoflurane anesthesia group were exposed to 1.5–2.5% sevoflurane and 100% O_2_ for 6 h. Mice anesthetized with sevoflurane were exposed to 3% sevoflurane and 60% O_2_ for 6 h. All prefrontal cortex tissues of marmosets and mice were harvested for the analysis of lipidomics.

**Results:**

Compared to the control group, we found that phosphatidylethanolamine (PE) (18:0/22:5), PE (16:0/22:5), PE (18:2/22:5), PE (14:0/22:5), and PE (18:1/22:5) increased in the prefrontal cortex of marmosets in the sevoflurane group, while triglyceride (TAG)56:5-fatty acid (FA) 20:4, TAG58:10-FA22:6, and TAG60:10-FA22:6 decreased. For aged mice, we indicated that lipid components phosphatidic acid (PA) (18:1/20:2) and TAG52:5-FA20:4 in the sevoflurane group increased, but PE (14:0/22:4), diglyceride (DAG) (16:1/18:2), and lysophosphatidylcholine (LPC) (16:1) + AcO decreased. More deeply, sevoflurane anesthesia resulted in the presence of 70 specific lipids in mice and marmosets. The enriched lipid subclasses were mainly monoacylglycerophosphoethanolamines and five other subclasses.

**Conclusion:**

Sevoflurane caused slight changes in lipid metabolism both in the aged brain of marmosets and mice. However, the pathways of lipid metabolism were not affected. The effects of sevoflurane on lipid metabolism in aged brains may differ among species.

## Introduction

Sevoflurane, an inhalation anesthetic, is widely used in general anesthesia. One of the central theories is that lipophilic sevoflurane could disrupt the fluidity, viscosity, and thickness of lipid rafts (domains of ordered lipids), and directly activates or inhibits signaling pathways by altering their lipid environment (Lerner, 1997; Weinrich and Worcester, 2018; Pavel et al., 2020). Abnormal lipid metabolism and neuronal damage have been demonstrated in the brains of infant monkeys after prolonged sevoflurane exposure (Liu et al., 2015). In addition, various types of lipids in the brain are a progressive detriment with age, especially after the age of 50 years old (Naudí et al., 2015). Therefore, assessing whether and/or how sevoflurane affected lipids in aged brains has become a focus of our attention.

Lipids are mainly known for their role in energy storage. However, thousands of lipids are involved in many important cellular functions *in vivo*, such as membranes constitution and compartmentalization, proteins stabilization and regulation, transport of cells, neurogenesis, synaptic transmission, regulation of gene expression, and cell signal transduction (Yang and Han, 2016; Wong et al., 2017). It has been reported that the brain is one of the most fat-rich organs in the human body, especially the lipid content (Cermenati et al., 2015). In nerve cells, lipids make up 50–60% of the components of the cell membrane (Cermenati et al., 2015). Therefore, lipids play an irreplaceable role in maintaining the normal operation of the nervous system. With the development of genomics, metabolomics, and molecular biology, abnormal lipid metabolism is involved in the regulation of many neurological diseases, including neurodegenerative diseases (Han, 2010), schizophrenia (Sabherwal et al., 2016), depression (Santos et al., 2021), and multiple sclerosis (Zhou et al., 2020), through oxidative stress, mitochondrial damage, myelin damage, inflammation, apoptosis, and other signal pathways (Wang et al., 2018a). Therefore, measuring lipid homeostasis in different physiological and pathological states can help us understand the underlying mechanisms and discover potential biomarkers for early diagnosis and prevention.

Recently, studies found an association between changes in lipids and age-related neurological diseases, such as Alzheimer’s disease (Zhan et al., 2014) and multiple system atrophy (Don et al., 2014). Owing to ethical restrictions and the lack of access to the disease-free human brains, there were few studies related to sevoflurane on lipid metabolism in the aged brain. Over millions of years of evolution, non-human primates share similar anatomy, genetic material, and physiological structures with humans, making them ideal models for considering human brain function (Ackermann et al., 2014). Therefore, compared with rodents, studies in non-human primates greatly simulated sevoflurane effects on lipid metabolism in the human brain (Okano et al., 2012). In the study, we analyzed the differences in lipids and lipid metabolic pathways between different species. We aimed to find the alterations of lipids after long-term sevoflurane anesthesia to determine the effects of sevoflurane on aged brains.

## Materials and Methods

All animal experiments were conducted in accordance with the NIH (the National Institutes of Health) guidelines for the care and use of laboratory animals.

### Marmosets

The marmoset research was performed according to the guidelines and regulations of the Institute of Animal Care Committee of the Center for Excellence in Brain Science and Intelligence Technology [CEBSIT, license NO. SYXK (Shanghai) 2021-0003], Chinese Academy of Sciences. This study was approved by the Institutional Animal Care and Use Committee of the Institute of Experimental Animal Science (protocol no. CEBSIT-2021035). In order to minimize the number of animals in the studies, six marmosets (*Callithrix jacchus*, 2 females and 4 males, 230–290 g) aged over 8 years were individually housed in a temperature (25–27°C) and humidity (30–70%)-controlled facility (12 h light/dark cycles) and supplied with *ad libitum* water and a balanced diet.

Three marmosets (1 female and 2 males) were assigned to the sevoflurane anesthesia group. These marmosets received 6–8% sevoflurane and 100% oxygen (2 L/min) for anesthesia induction (1–2 min), followed by 1.5–2.5% sevoflurane and 100% oxygen (2 L/min) for maintenance of anesthesia. They were inhaled with sevoflurane through mask ventilation without intubation (Coleman et al., 2017; Walters et al., 2019). Three other marmosets were included in the control group (1 female and 2 males). The marmosets in the control group were only anesthetized with 6–8% sevoflurane and 100% oxygen (2 L/min) for anesthesia induction for 1–2 min. The vital signs of marmosets were monitored by Monitors (BeneVision M12, Mindray, China) during general anesthesia, and a warm blanket (AHM06, Reptizoo, China) was given to keep the temperature at 37 ± 0.5°C. At the end of sevoflurane anesthesia, marmosets were mercy sacrificed under deep anesthesia with 3–5% sevoflurane. Before sacrifice, blood was drawn from the femoral artery for blood gas analysis of vital signs using a portable clinical analyzer (i-STAT; Abbott Laboratories Inc., East Windsor, NJ, United States). Characteristics and results of blood gases analysis of the marmosets are shown in Supplementary Table 1. The prefrontal cortex of all marmosets was collected quickly and frozen in liquid nitrogen for the lipid metabolism.

### C57/6J Mice

All C57/6J mice aged 18 months were provided by Beijing Vital River Laboratory Animal Technology Co., Ltd., Beijing, China in our study. The animal care protocol (SH9H-2021-A831-1) was approved by the Institutional Animal Care and Use Committee of Shanghai Ninth People’s Hospital. Ten mice were housed in a temperature- and humidity-controlled facility with an indoor temperature of 25°C and 12 h light/dark cycles. The animals were given free access to food and water.

A total of five aged mice were anesthetized with 3% sevoflurane and 60% O_2_ (1 L/min) for 6 h. Another five aged mice in the control group received 60% O_2_ (1 L/min) for 6 h. The mice were put in an anesthetizing chamber. The rectal temperature of mice was maintained at (37 ± 0.5)°C by the incubator. All mice were mercy sacrificed with CO_2_. Before sacrifice, the arterial blood gas values were detected. The values of pH, PaO_2_, and PaCO_2_ were 7.34 ± 0.05, 178.7 ± 46.62, and 45.5 ± 4.02, respectively, and there were no differences between the sevoflurane and control groups. The prefrontal cortex of 10 mice was quickly taken and frozen in liquid nitrogen for the experiment on lipid metabolism.

### Analysis of Liquid Chromatography–Tandem Mass Spectrometry

The liquid chromatography–tandem mass spectrometry (LC-MS/MS) analysis was performed with Agilent 1290 Infinity II system (Agilent Technologies, United States) coupled with QTRAP 6500+ triple quadrupole linear ion trap hybrid MS system (AB SCIEX Technologies, United States). Chromatographic separation was achieved at 40°C with an ACQUITY UPLC BEH C18 column (2.1 × 100 mm, 1.7 μm) (Waters Technologies, United States).

Reversed-phase chromatography was used for the separation and analysis of sample extracts. The mobile phase was composed of solvent A water (containing 30% methyl alcohol and 30% ACN, v/v) and solvent B (IPA) at a constant flow rate of 0.35 ml/min. The temperature of the autosampler was 10°C. The injection volume was 2 μl, and the total run time for the analysis of a single sample was 2 min.

MS detection was performed in positive ion mode, using multiple reaction monitoring (MRM) mode. MS system operated in both positive and negative ionization modes and was controlled by the Analyst 1.7.2 software (AB SCIEX Technologies, United States). Operational parameters of MS analysis were as follows: curtain gas at 35 psi, nebulizing gases GS1 at 50 psi and GS2 at 55 psi, collision gas with nitrogen at medium, positive mode ion spray voltage at 5500 V, negative mode ion spray voltage at −4500 V, Jet Stream electrospray ionization (ESI) source operating temperature at 450°C. Data were processed with OS V 2.1 software (AB SCIEX Technologies, United States). All mass spectrometer conditions were optimized for quantitative detection of the analytes.

### Sample Preparation

Quantification was done by external calibration with internal standards. Standard working solutions were prepared by diluting 10-fold the lipid standard working solutions with alcohol. Prefrontal cortex homogenate standards were prepared in blank mice and marmosets prefrontal cortex homogenate by spiking the diluted standard working solutions. A 20 μl aliquot of prefrontal cortex homogenate was mixed with the diluted standard working solutions and 750 μl methyl tertiary butyl ether. The sample was vortexed for approximately 10 s and let stand for a period of 30 min, and then 200 μl pure water-MS grade was added. After centrifugation at 15,000 rpm for 15 min at 4°C, 700 μl of the supernatant was transferred into a new centrifuge tube. The supernatant was evaporated to dryness with a stream of nitrogen at room temperature. The dry residue was reconstituted with 100 μl of dichloromethane and methanol (1:1, v/v), and transferred to autosampler vials.

### Statistical Analysis

A multivariate (dimensional) statistical analysis was conducted using the MetaboAnalyst 5.0 online analysis tool.^1^ Prior to the multi-dimensional statistical modeling, an autoscaling was used for standardization. An unsupervised principal component analysis (PCA) was used for the analysis of metabolomics raw data to assess the specificity of lipids and metabolic profiles. The partial least squares discriminant analysis (PLS-DA) established the relationship mode between metabolite expression and sample categories to assist in the screening of lipids. Metabolic phenotypic variances corresponding to the classes were received by using supervised orthogonal projection to latent structure discriminant analysis (orthogonal partial least squares discriminant analysis, OPLS-DA). The variable importance in projection (VIP) values represented the effect of variables on the formation of vectors. The variables with VIP value > 1 indicated that the influence of the Y matrix was higher than the average level. The lipids that met both the OPLS-DA analysis screening criteria (VIP > 1) and the volcano plot analysis screening criteria (*P* < 0.05) were regarded as lipids with significant differences. WGCNA (version 1.70-3 in R studio) completed the search for gene modules of cooperative expression and analyzed the relationship between lipids. Through the dimensionality reduction of high-dimensional lipidome data, the composition of lipids in different gene modules was further counted. Lipid expression profile data were submitted to the BioPAN online analysis tool^2^ for lipid pathway analysis. The *Z* value based on reaction weights was used to determine whether pathway reactivity was inhibited in a group of samples.

In addition, the Student’s *t*-tests were performed using the GraphPad Prism 6.0 software to measure the significance of each metabolite. The data were presented as means ± SD. The significance was fixed at *P* < 0.05.

## Results

### Sevoflurane Induced Alterations of Lipids in the Prefrontal Cortex of Aged Marmosets

**FIGURE 1:**
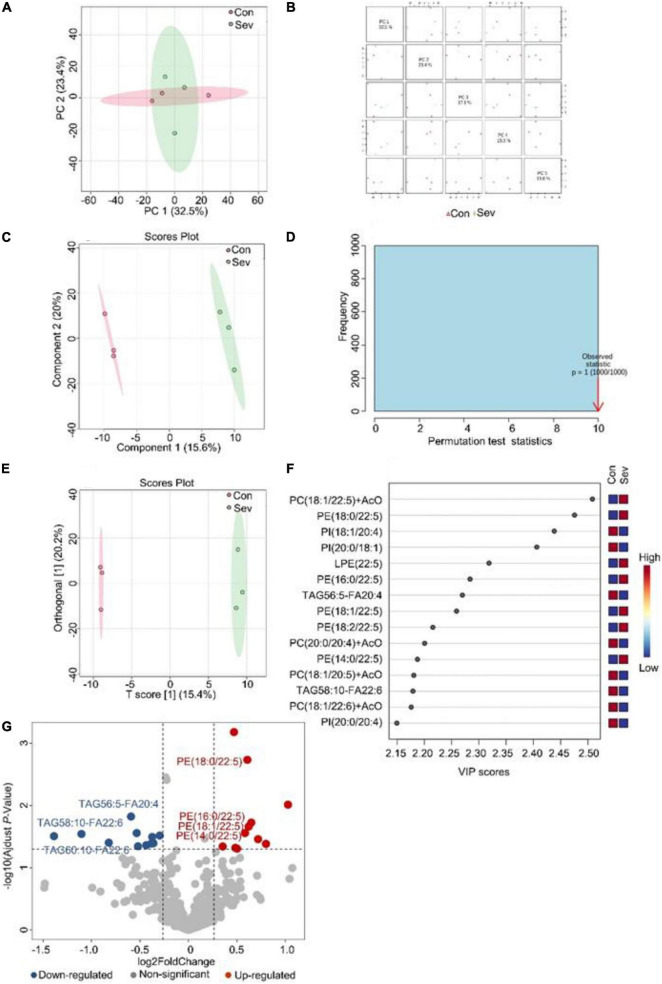
Analysis of lipidomics in the prefrontal cortex of aged marmosets. **(A)** Two-dimensional score diagram of PCA analysis of sevoflurane anesthesia group and control group in prefrontal cortex of aged marmosets. **(B)** Pairwise score chart of the top five principal components of PCA. **(C)** PLS-DA 2D score chart. **(D)** Permutation test results of PLS-DA Model 1000 times. **(E)** OPLS-DA 2D score chart. **(F)** The top 15 lipids ranked by VIP value of the OPLD-DA model. **(G)** In volcano analysis, red dots indicated the lipid components significantly increased in the sevoflurane group compared with the control group (fold-change > 1.2, *P* < 0.05); blue dots indicated obviously lower lipid components in the sevoflurane group (fold-change < 0.83, *P* < 0.05).

To visualize the differences in lipids, an unsupervised pattern discriminant analysis was performed using PCA (Figures 1A,B). Score plots showed that the separation trend was not obvious in the control group and sevoflurane group. There were in component 1 (15.6%) and component 2 (20%) for PLS-DA (Figure 1C). These results indicated a significant difference in lipids in the prefrontal cortex of marmosets after sevoflurane anesthesia. However, the result of 1000 permutation tests of the PLS-DA model showed that the *P*-value was 1 (Figure 1D), demonstrating that the PLS-DA model may have been overfitting. As shown in Figures 1E,F, the VIP scores were also performed to identify the lipids that contributed most significantly to the effect of sevoflurane, based on the OPLS-DA model of metabolites data of marmosets. Fifteen lipids were found to be significantly different (VIP score) between the two groups. Compared with the control group, concentrations of seven lipid components [phosphatidylcholine (PC), phosphatidylethanolamine (PE), and lysophosphatidylethanolamine] were higher in the sevoflurane group, but those of eight lipid components [phosphatidylinositol (PI), PC, and triglyceride (TAG)] decreased. The volcano plot analysis revealed that PE (18:0/22:5), PE (16:0/22:5), PE (18:2/22:5), PE (14:0/22:5), and PE (18:1/22:5) increased in the sevoflurane group. The TAG56:5-fatty acids (FAS) 20:4, TAG58:10-FA22:6, and TAG60:10-FA22:6 decreased (Figure 1G). We selected lipids that met both the OPLS-DA analysis screening criteria (VIP > 1) and volcano plot analysis screening criteria (*P* < 0.05) as remarkably different lipids (Table 1).

**TABLE 1:** Differential lipids in marmosets.

Lipid	Fold-change	Log_2_Fold-change	*P*-value	VIP
PC (18:1/22:5) + AcO	1.385	0.470	0.001	2.508
PE (18:0/22:5)	1.524	0.608	0.002	2.476
PI (18:1/20:4)	0.850	–0.235	0.004	2.438
PI (20:0/18:1)	0.855	–0.226	0.004	2.406
LPE (22:5)	2.038	1.027	0.010	2.319
TAG56:5-FA20:4	0.663	–0.593	0.015	2.270
PE (16:0/22:5)	1.568	0.649	0.019	2.284
PE (18:1/22:5)	1.539	0.622	0.022	2.259
PE (18:2/22:5)	1.499	0.584	0.028	2.216
PC (20:0/20:4) + AcO	0.691	–0.534	0.028	2.200
TAG58:10-FA22:6	0.465	–1.104	0.029	2.179
PC (18:1/22:6) + AcO	0.814	–0.297	0.030	2.176
PC (18:1/20:5) + AcO	0.382	–1.388	0.031	2.181
PI (20:0/20:4)	0.772	–0.374	0.032	2.149
PE (16:0/18:1)	1.124	0.169	0.034	2.121
PE (14:0/22:5)	1.646	0.719	0.035	2.187
TAG60:10-FA22:6	0.566	–0.822	0.040	2.127
PE (P-18:2/22:6)	0.778	–0.362	0.040	2.104
PC (20:0/16:1) + AcO	0.766	–0.385	0.041	2.138
TAG54:2-FA20:0	1.741	0.800	0.041	2.077
TAG56:7-FA22:6	0.739	–0.436	0.043	2.127
TAG54:6-FA22:6	0.697	–0.521	0.045	2.116
PI (16:0/20:2)	1.278	0.354	0.046	2.106
PA (20:0/22:5)	1.399	0.484	0.048	2.113
DAG (16:0/22:5)	1.416	0.502	0.049	2.065

*The lipids that met both OPLS-DA analysis screening criteria (VIP > 1) and volcano plot analysis screening criteria (P < 0.05) were selected as the lipid components with significant difference.*

### Sevoflurane Induced Alterations of Lipids in the Prefrontal Cortex of Aged Mice

**FIGURE 2:**
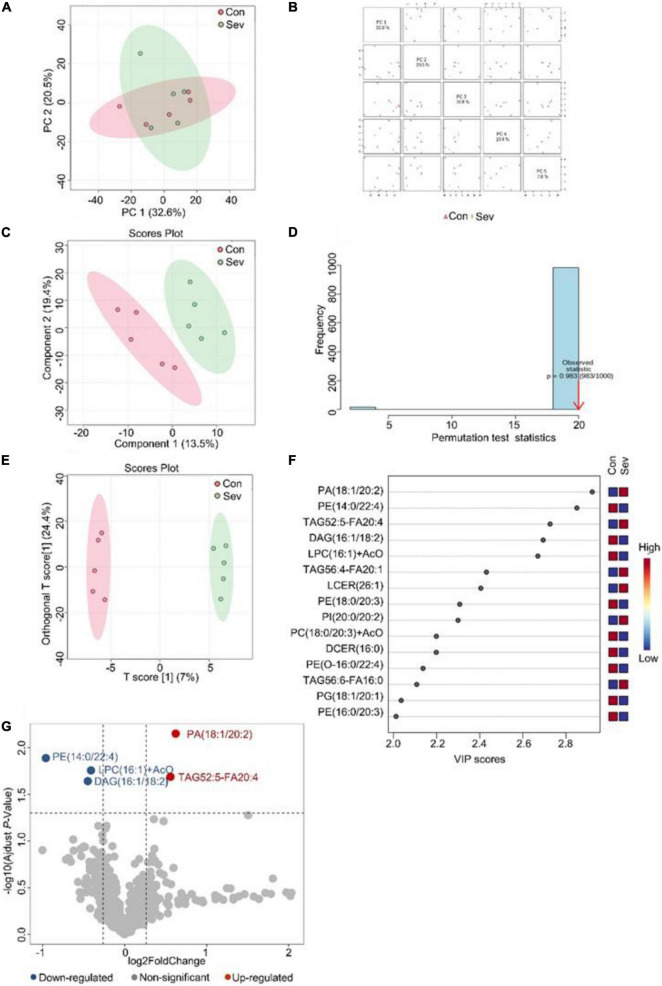
Analysis of lipidomics in the prefrontal cortex of aged mice. **(A)** Two-dimensional score diagram of PCA analysis of sevoflurane anesthesia group and control group in prefrontal cortex of aged mice. **(B)** Pairwise score chart of the top five principal components of PCA. **(C)** PLS-DA 2D score chart. **(D)** Permutation test results of PLS-DA Model 1000 times. **(E)** OPLS-DA 2D score chart. **(F)** The top 15 lipids ranked by VIP value of the OPLD-DA model. **(G)** In volcano analysis, red dots indicated the lipid components significantly increased in the sevoflurane group compared with the control group (fold-change > 1.2, *P* < 0.05); blue dots indicate significantly lower lipid components in the sevoflurane group (fold-change < 0.83, *P* < 0.05).

Similarly, an unsupervised pattern discriminant analysis was performed by PCA (Figures 2A,B). Score plots cleared that the separation trend was not obvious in the control and sevoflurane groups. There were in component 1 (13.5%) and component 2 (19.4%) for PLS-DA (Figure 2C). The result of 1000 permutation tests of the PLS-DA model showed that the *P*-value was 0.983 (Figure 2D) and uncovered that the PLS-DA model might be overfitting. The results demonstrated a significant difference in lipids in the prefrontal cortex of mice after sevoflurane anesthesia. However, OPLS-DA separated the samples into two colonies (Figure 2E). As shown in Figure 2F, the VIP scores were also performed to identify the lipids that contributed most significantly to the effect of sevoflurane, which is based on the OPLS-DA model of metabolites data of mice. Fifteen lipids were found to be significantly different (VIP score) between the two groups. Compared with the control group, concentrations of six lipid components [phosphatidic acid (PA), TAG, PI, and lactosyl ceramide] were higher in the sevoflurane group and those of nine lipid components [diglyceride (DAG), PE, lysophosphatidylcholine (LPC), PC, and phosphatidylglycerol] decreased. The volcano plot analysis showed that PA (18:1/20:2) and TAG52:5-FA20:4 increased in the sevoflurane group. PE (14:0/22:4), DAG (16:1/18:2), and LPC (16:1) + AcO decreased (Figure 2G). We selected lipids that met both the OPLS-DA analysis screening criteria (VIP > 1) and volcano plot analysis screening criteria (*P* < 0.05) as significantly different lipids (Table 2).

**TABLE 2:** Differential lipids in mice.

Lipid	Fold-change	Log_2_Fold-change	*P*-value	VIP
PA (18:1/20:2)	1.538	0.621	0.007	2.922
PE (14:0/22:4)	0.514	–0.961	0.013	2.850
LPC (16:1) + AcO	0.752	–0.411	0.018	2.669
TAG52:5-FA20:4	1.473	0.558	0.021	2.726
DAG (16:1/18:2)	0.732	–0.450	0.023	2.694

*The lipids that meet both OPLS-DA analysis screening criteria (VIP > 1) and volcano plot analysis screening criteria (P < 0.05) were selected as the lipid components with significant difference.*

### Analysis of Lipid Metabolic Pathways in the Prefrontal Cortex of Marmosets and Mice

**FIGURE 3:**
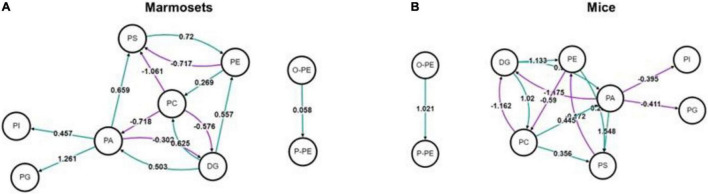
Analysis of lipid metabolic pathways in marmosets and mice. **(A)** Lipid metabolic pathways in marmosets. **(B)** Lipid metabolic pathways in mice.

We used BioPAN to study all possible lipid synthesis pathways from the Reactome database associated with changes in lipid metabolism after long-term sevoflurane anesthesia. But we did not find lipid metabolic pathways inhibited and activated in the prefrontal cortex of marmosets and mice (Figure 3).

### Differences in Prefrontal Cortex Lipids Between Marmosets and Mice in Control Group and Sevoflurane Group

**FIGURE 4:**
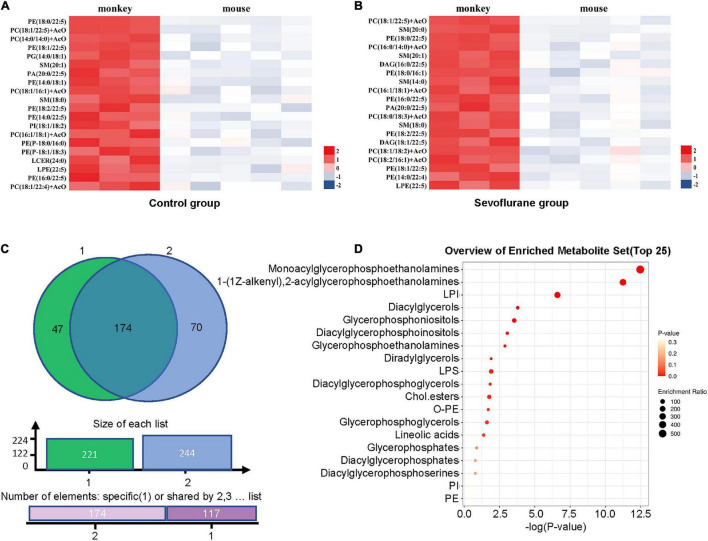
Analysis of lipid differences between marmosets and mice in control group and sevoflurane group. **(A)** Heat map of differential lipids in the top 20 of control groups between marmosets and mice. **(B)** Heat map of differential lipids in the top 20 of sevoflurane groups between marmosets and mice. **(C)** Venn plot analysis. **(D)** Subclass enrichment analysis.

The lipids that met the screening criteria of OPLS-DA analysis (VIP > 1) and volcano plot analysis (*P* < 0.05) were selected as those with significant differences between marmosets and mice. The heat maps of differential metabolite expression in the top 20 of control group and sevoflurane group were displayed in Figures 4A,B (all differential lipids between marmosets of control group and sevoflurane group were shown in Supplementary Table 2), respectively. Through Venn plot analysis, we found that there were 221 differential lipids in control group and 244 in sevoflurane group. Further analysis demonstrated that a total of 174 different lipids were present in marmosets and mice in both groups (Figure 4C). Subclass enrichment analysis of common differential lipids in sevoflurane group showed that after anesthesia, 70 specific differential lipids (Figure 4C) were enriched in 19 lipid subclasses (Figure 4D). The significantly enriched lipid subclasses were monoacylglycerophosphoethanolamines and five other subclasses [false discovery rate (FDR) < 0.05].

## Discussion

Lipids are important substances in the brain, performing signaling processes related to the cell cycle, stress, and inflammation to help maintain brain and cellular homeostasis (Frisardi et al., 2011; Zhang and Liu, 2015). It has been reported that inhalation anesthetics disrupted lipid rafts on membranes, which were key factors in the signaling process of cellular responses (Pavel et al., 2020). However, there is currently a lack of direct evidence to prove the effects of anesthetics on the lipid metabolism of the brain. In this study, lipidomics analysis of the prefrontal cortex was discussed based on aged marmosets and mice anesthetized with sevoflurane for 6 h. Multivariate statistical methods (PCA, PLS-DA, and OPLS-DA) were used to analyze lipid profiles and screen differential lipids. Long-term sevoflurane anesthesia was confirmed to induce slight alterations of lipids in the prefrontal cortex, rather than lipid metabolic pathways. Moreover, there were species differences in this change.

With the widespread and growing use of general anesthesia, sevoflurane has become a popular anesthetic for elderly patients. It is important to study the potential adverse effects of sevoflurane on lipid metabolism. PE, a key lipid component, is located on the inner side of the plasma membrane and is abundant in the brain (Kim et al., 2010). In the analysis of lipidomic, we found that PE in the prefrontal cortex of aged marmosets significantly increased after long-term anesthesia with sevoflurane, suggesting changes in phospholipid metabolism and content. Interestingly, the transformation of intracerebral PE in infant rhesus monkeys after long-term exposure to sevoflurane (2.5%, 9 h) was contrary to our data (Liu et al., 2015). This was presumably due to differences in lipid metabolism pathways at different periods from infancy to old age. Long-term exposure to sevoflurane could disrupt the homeostasis of phospholipids in the brain, and change the integrity, permeability, and direction of the plasma membrane, which may cause damage to the nervous system (Wang et al., 2018b; Mesa-Herrera et al., 2019). We also found that TAG in the brain of aged marmosets decreased. TAG, an important structural component of the nerve cell membrane, not only ensures the integrity of membrane structure but also acts as a signal molecule to provide energy to cells (Mesa-Herrera et al., 2019). Due to the supply of sugar and the amino acids being insufficient during sevoflurane anesthesia, lipid decomposition, and energy supply may initiate the decrease of TAG.

The change of lipids is diversified in the cerebral cortex of marmosets and mice, including PE, TAG, PA (modulating excitability and pain-related ion channels) (Robinson et al., 2019), DAG, and LPC (regulating demyelination) (Plemel et al., 2018). Surprisingly, the expression of PE and TAG in the brain of aged mice after anesthesia was opposite to those in aged marmosets. Therefore, in biomedical research, the regulation mechanism of mice simulating human physiology and pathology was debatable, which was also the common view of many scholars (Coleman et al., 2017; Raper et al., 2021).

Differences in lipids after anesthesia were associated with neurotoxicity-induced systemic energy deficits (Wang et al., 2018a) and neuronal apoptosis (Groc et al., 2001) in developing brains. The changes in lipid metabolism are related to age (Naudí et al., 2015), and the effects of anesthetics such as sevoflurane on the aging brain are also related to the increase in age (Liu et al., 2015). Meanwhile, lipid metabolism has been implicated in brain disease in the elderly (Don et al., 2014; Zhan et al., 2014). Elderly patients are more likely to develop POCD and delirium after sevoflurane anesthesia (Tang et al., 2014; Qiao et al., 2015), which may be related to the effect of sevoflurane on brain metabolism. In the present study, prolonged exposure to sevoflurane caused slight alterations in lipid metabolism in the cerebral cortex of both aged marmosets and mice. However, none of their lipid classification modules were markedly different using WGCNA analysis (Supplementary Figure 1). Meanwhile, lipid signaling pathways were neither activated nor inhibited. It evidenced that changes in lipids could not cause variations in signal pathways.

For the elderly marmosets were more precious, we only conducted three-to-three anesthesia experiments. If the sample size was too small, there may be an over-fitting phenomenon, which needs to be further confirmed. If possible, after further increasing the sample size, the study should be repeated and analyzed for the lipid metabolism at multiple time points. We could further explore the hidden mechanisms of anesthesia on lipid metabolism and the signaling pathways in nuclei of the aged brain with larger cohorts.

## Conclusion

Sevoflurane caused slight changes in brain lipid metabolism (PA, PC, PE, PI, and TAG) both in aged marmosets and mice. However, the lipid metabolic pathways were not affected. In addition, the effects of sevoflurane on lipid metabolism in aged brains may differ among species.

## Data Availability Statement

The original contributions presented in the study are included in the article/Supplementary Material, further inquiries can be directed to the corresponding authors.

## Ethics Statement

The marmoset research was performed according to the guidelines and regulations of the Institute of Animal Care Committee of the Center for Excellence in Brain Science and Intelligence Technology [CEBSIT, license NO. SYXK (Shanghai) 2021-0003], Chinese Academy of Sciences. This study was approved by the Institutional Animal Care and Use Committee of the Institute of Experimental Animal Science (protocol No. CEBSIT-2021035).

## Author Contributions

ZQ and HJ: conceptualization. HM, YC, LS, XC, RZ, ZX, and SL: methodology. HM, JZ, and ZQ: writing – original draft preparation. HM, ZQ, and HJ: writing – review and editing. All authors contributed to the article and approved the submitted version.

## Conflict of Interest

The authors declare that the research was conducted in the absence of any commercial or financial relationships that could be construed as a potential conflict of interest.

## Publisher’s Note

All claims expressed in this article are solely those of the authors and do not necessarily represent those of their affiliated organizations, or those of the publisher, the editors and the reviewers. Any product that may be evaluated in this article, or claim that may be made by its manufacturer, is not guaranteed or endorsed by the publisher.

## Footnotes

1
https://www.metaboanalyst.ca/

2
https://lipidmaps.org/biopan/load_data.php/

## Supplementary Material

Supplementary Figure 1 WGCNA analysis of marmosets and mice. **(A)** A total of 632 lipid components of marmosets were aggregated into 12 modules. Module 11: *P* < 0.05. **(B)** A total of 632 lipid components of mice were aggregated into 11 modules. Statistical analysis of the modules showed that *P* > 0.05.
