# Supplementary material for: Effects of Sevoflurane Anesthesia on Cerebral Lipid Metabolism in the Aged Brain of Marmosets and Mice
Source: Front Mol Neurosci. 2022 Jul 6;15:915570. doi: 10.3389/fnmol.2022.915570 (PMC9298509; doi:10.3389/fnmol.2022.915570)
Supplement: Supplementary file 1 [file Table_1.doc]

**SUPPLEMENT TABLE 1** Baseline characteristics of aged marmosets and aged mice

|  | **Control group**  **(n=3)** | **sevoflurane anesthesia group**  **(n=3)** | ***P* value** |
| --- | --- | --- | --- |
| Age (yr) | 8.33 (0.577) | 8.67 (0.577) | 0.519 |
| Sex | 2M,1F | 2M,1F |  |
| Weight (g) | 261.67 (28.01) | 286 (2.00) | 0.208 |
| HR (bmp min^-1^) | 185.00 (5.29) | 189.33 (4.04) | 0.323 |
| RR (r min^-1^) | 45.33 (4.73) | 45.67 (3.06) | 0.923 |
| T (℃) | 37.67 (0.252) | 36.93 (0.29) | 0.073 |
| SPO2 (%) | 99.67 (0.58) | 98.67 (1.15) | 0.251 |
| pH | 7.44 (0.09) | 7.57 (0.10) | 0.165 |
| PaO2 (kPa) | 39.95 (25.20) | 35.07 (3.05) | 0.756 |
| PaCO2 (kPa) | 6.37 (1.36) | 5.22 (1.16) | 0.329 |
| BE (ecf) (mmol L^-1^) | 8.67 (11.02) | 13.33 (4.04) | 0.529 |
| HCO3^-^ (mmol L^-1^) | 32.90 (9.70) | 35.43 (3.65) | 0.694 |
| TCO2 (mmol L^-1^) | 34.33 (10.02) | 36.67 (3.79) | 0.725 |
| SO2 (%) | 99.67 (0.58) | 100.00 (0.00) | 0.423 |

Continuous variables are median (standard error of the mean). HR, heart rate; RR, respiratory rate; T, body temperature; SPO2, transcutaneous oxygen saturation; pH, Arterial pH; PaO2, arterial partial pressure of oxygen; PaCO2, arterial partial pressure of carbon dioxide; BE (ecf), arterial blood actual alkali reserve or alkali surplus; TCO2, Total carbon dioxide; SO2, oxygen saturation.
