# Supplementary material for: Effects of Sevoflurane Anesthesia on Cerebral Lipid Metabolism in the Aged Brain of Marmosets and Mice
Source: Front Mol Neurosci. 2022 Jul 6;15:915570. doi: 10.3389/fnmol.2022.915570 (PMC9298509; doi:10.3389/fnmol.2022.915570)
Supplement: Supplementary file 2 [file Table_2.doc]

**SUPPLEMENT TABLE 2A** Lipid Metabolomic characteristics of prefrontal cortex between aged marmosets and mice in control group.

VIP, variable importance in projection scores.

|  | **Marmoset** | **Marmoset** | **Marmoset** | **Mice** | **Mice** | **Mice** | **Mice** | **Mice** | **raw.pval** | **VIP** |
| --- | --- | --- | --- | --- | --- | --- | --- | --- | --- | --- |
| PE(18:0/22:5)  PC(18:1/22:5)+AcO  PC(14:0/14:0)+AcO  PE(18:1/22:5)  PG(14:0/18:1)  SM(20:1)  PA(20:0/22:5)  PE(14:0/18:1)  PC(18:1/16:1)+AcO  SM(18:0)  PE(18:2/22:5)  PE(14:0/22:5)  PI(18:1/18:2)  PC(16:1/18:1)+AcO  PE(P-18:0/16:0)  PE(P-18:1/18:3)  LCER(24:0)  LPE(22:5)  PE(16:0/22:5)  PC(18:1/22:4)+AcO  PE(P-18:0/18:3)  PC(14:0/18:1)+AcO  PE(14:0/22:4)  DAG(18:1/22:5)  PC(20:0/22:4)+AcO  SM(20:0)  PE(16:0/16:1)  PE(18:0/16:1)  LCER(26:0)  PE(O-16:0/18:3)  DAG(16:0/22:6)  PI(18:0/22:4)  PE(18:1/18:3)  PC(16:0/14:0)+AcO  PI(16:0/22:4)  PE(18:0/16:0)  PE(O-18:0/16:0)  PC(16:0/16:1)+AcO  SM(14:0)  PE(O-16:0/16:1)  PC(16:0/18:3)+AcO  PC(16:1/18:2)+AcO  TAG48:0-FA16:0  PC(18:0/18:3)+AcO  PE(O-18:0/16:1)  PE(O-18:0/22:4)  LCER(24:1)  TAG54:6-FA22:6  PI(18:1/22:4)  PG(18:0/18:1)  PE(O-16:0/18:2)  PC(18:0/14:0)+AcO  DAG(16:0/22:5)  PI(18:0/20:3)  SM(16:0)  LCER(26:1)  PC(18:1/18:2)+AcO  CER(14:0)  PE(18:2/20:2)  PI(18:0/20:4)  PE(14:0/20:4)  PC(18:0/16:1)+AcO  PC(18:0/18:2)+AcO  PE(P-18:0/16:1)  PC(18:1/20:2)+AcO  LCER(22:0)  PE(P-18:0/22:4)  PG(18:0/22:4)  PG(18:1/18:2)  PE(18:1/22:4)  DCER(26:1)  PC(18:2/16:1)+AcO  PG(18:1/16:1)  PG(18:0/18:2)  SM(26:0)  PC(18:1/18:3)+AcO  PE(18:0/22:4)  PI(20:0/20:4)  PG(18:1/18:1)  PC(16:0/22:5)+AcO  PE(P-16:0/22:6)  PE(16:0/14:0)  PA(20:0/22:4)  PE(18:0/18:2)  LPC(22:6)+AcO  PE(O-16:0/20:4)  FFA(18:3)  PE(O-18:0/20:4)  PG(18:2/18:2)  DAG(16:0/20:4)  PE(O-18:0/18:2)  PC(16:0/18:2)+AcO  PE(O-16:0/22:4)  PE(16:0/20:2)  PC(18:2/20:2)+AcO  PC(18:2/18:2)+AcO  PE(18:2/20:4)  LPC(16:0)+AcO  LCER(20:0)  PI(18:1/20:3)  PE(18:0/18:0)  PI(20:0/16:1)  PI(16:0/20:4)  DCER(18:0)  PE(18:0/18:3)  SM(26:1)  PE(14:0/22:6)  LPE(16:0)  PG(18:1/22:4)  TAG58:10-FA20:4  HCER(26:0)  PE(18:0/20:2)  PE(18:2/18:2)  TAG56:7-FA18:1  PE(P-18:1/16:0)  DCER(20:0)  PG(16:0/18:2)  TAG56:7-FA16:0  PC(18:0/22:4)+AcO  PG(18:1/20:2)  PI(20:0/18:2)  DAG(16:0/16:0)  HCER(26:1)  PI(18:1/16:1)  PA(18:1/18:2)  PE(16:0/22:4)  DCER(24:0)  PG(16:0/18:0)  PC(16:0/20:4)+AcO  PG(16:0/18:1)  TAG50:0-FA16:0  PI(18:1/18:1)  PC(18:2/20:3)+AcO  DAG(18:1/22:6)  SM(24:0)  PG(18:0/18:0)  PI(18:1/20:4)  PC(18:2/20:1)+AcO  PI(18:1/20:2)  PE(18:1/20:2)  PC(18:1/20:3)+AcO  PA(20:0/18:2)  LPC(20:4)+AcO  TAG54:6-FA16:0  PI(18:0/18:0)  PC(16:0/22:4)+AcO  PA(20:0/16:1)  TAG50:0-FA18:0  PC(14:0/18:2)+AcO  TAG56:5-FA22:4  PG(16:0/20:4)  PI(18:0/18:2)  PE(18:0/20:3)  SM(18:1)  PG(18:0/20:2)  PE(P-16:0/18:3)  PI(18:0/22:5)  PE(P-18:0/18:2)  PI(16:0/20:2)  PI(16:0/18:2)  LPC(18:1)+AcO  PA(20:0/20:4)  PE(18:1/18:2)  PG(16:0/22:4)  PE(18:1/18:1)  PG(18:1/22:6)  PE(16:0/18:2)  PC(18:2/20:4)+AcO  CER(20:1)  PS(16:0/18:0)  PG(18:0/16:1)  TAG56:7-FA22:6  PG(16:0/16:0)  CE(20:4)  LPI(16:0)  LPC(14:0)+AcO  LPC(18:0)+AcO  PA(18:1/20:2)  TAG56:7-FA22:5  DAG(18:2/20:4)  LPE(18:0)  PC(18:1/20:5)+AcO  PE(P-18:1/18:2)  DCER(22:0)  PE(P-18:0/20:4)  PE(P-18:2/20:4)  TAG50:1-FA18:1  PC(16:0/22:6)+AcO  PE(P-16:0/20:4)  PE(16:0/18:3)  PE(16:0/22:6)  LPC(20:0)+AcO  LPE(22:6)  PE(18:1/20:5)  PC(18:1/18:1)+AcO  PI(18:0/20:1)  PE(16:0/20:4)  PE(O-18:0/22:6)  PE(P-16:0/22:5)  DAG(16:0/18:0)  DAG(16:1/20:4)  PE(P-18:1/22:4)  PE(18:0/14:0)  PI(18:0/20:0)  PC(18:0/20:3)+AcO  TAG54:4-FA20:4  PE(P-18:2/18:2)  PE(16:0/20:5)  PI(20:0/18:1)  TAG50:1-FA16:0  TAG55:5-FA18:1  CE(18:2)  FFA(20:4)  LPC(16:1)+AcO  TAG44:0-FA18:0  PE(O-16:0/20:2)  TAG56:5-FA18:0  TAG58:10-FA22:6  PE(O-16:0/20:5)  PE(18:1/20:3)  DAG(16:0/20:3) | 15917966.94  290827.2917  127326.2809  1235711.468  44652.20966  5343919.961  1998603.41  232179.9989  3998060.654  680553628.9  60527.87993  15238.49606  79197.75457  3780976.342  16099228.2  43707.33318  1919292.405  99072.92999  2792081.834  276177.5737  62005.95492  2410778.243  27673.98548  135135.9917  51195.29484  281146186.9  854179.1546  680510.6198  161796.4783  21262.87214  1140154.374  453333.1902  70906.84614  6569208.996  252215.7302  2574659.142  2794930.549  16655977.9  1888487.739  158380.208  684660.4161  312978.0614  3414755.918  168060.3407  247303.4167  10721373.98  4324473.078  284758.5858  113777.0531  2268492.289  138419.0677  544709.9457  350866.1106  1562295.184  54813627.88  291987.7023  2146013.159  357185.326  33292.99496  26997473.86  23263.84298  2606526.489  5603062.05  237491.1706  227926.6101  75279.81888  72904267.14  239249.8099  64507.68713  2645926.136  78554.96228  191560.9249  190507.719  63247.19665  5821510.272  103749.7766  29709199.51  118064.8045  1428482.61  2205072.108  5120219.762  118582.5893  13804987.28  4722110.081  9165.642704  2135522.242  1651520.461  2954935.847  32716.43708  2806702.379  180668.6272  9976254.731  3586940.839  385283.0604  28804.43968  290107.7576  357879.7569  2027284.592  226434.5707  210497.4556  810781.3717  69092.75457  2553458.913  6898331.695  169134.6633  7683460.909  27252.15116  665477.7838  65475.99197  59362.35629  2052967.851  1340968.982  103195.8938  109499.3835  15278686.57  260444.4807  66138.40669  97722.54748  2295600.608  20390.06434  68223.46367  2124530.536  9308744.093  681497.0028  171934.0729  4264347.486  78533.89541  458466.6839  17502134.52  10061733.14  2687020.739  692904.9717  47156.4445  553279.1162  33962179.61  105742.5231  3473278.454  56693.43852  21999.16469  451531.8147  423665.4252  284100.5231  44565.38594  127207.4483  717736.9658  1673929.72  560255.2365  1620559.74  92472.92264  45592.54428  686463.2307  236350.4293  4910573.277  35267285.99  56381.29772  14106.7938  198427.5038  1043120.517  41468.00696  177696.5553  747374.869  8566523.516  1696421.937  43458.94673  24870950.54  6679.126731  1392449.9  466412.2382  4392358.466  414528.6164  41499.65329  525536.4068  1976950.512  205279.7592  17287.23881  27149.18684  1500823.876  15073.26913  30447.66779  78845.62949  4236571.187  22413.80157  672971.4009  46342.40951  30912460.76  348140.2923  2129269.762  7426053.007  4390109.41  45437.42461  9244936.259  5203.656107  324319.7013  21454.70129  17693307.63  47637.39526  5327790.78  3258322.64  570349.5079  1180708.026  53724.30501  21217562.94  39398.19249  1385484.477  1761869.729  165260.9706  31968.2211  4038.691212  945562.8528  3899033.287  23911.89782  99046.1415  54456738.17  61662.5287  81223.27544  163758.8157  35747.65343  82461.22852  14813.39219  999204.4469  139481.571 | 15900962.55  285325.8943  132730.687  1304696.264  52321.62471  5082842.188  1514672.887  208112.7815  3975008.757  799522750.9  71771.87253  11950.74267  74885.51071  3809681.556  13940467.97  56683.49142  1370695.786  74626.38271  2028474.488  308235.7922  68399.37997  2454839.886  30325.24901  94038.96783  65260.37349  271394452.2  783261.1698  656312.4639  109059.1766  15691.27758  722365.6829  533795.0193  83038.58982  5423930.887  233577.6389  2631507.81  2495565.697  17432711.06  2120144.948  124725.6062  923666.7439  381406.6255  2804815.477  243013.5566  284876.3736  13909820.96  2392460.937  281786.4008  78771.41915  2172458.648  110731.737  454043.6787  230458.738  1345739.975  75866857.7  148196.4889  2974495.118  388048.089  33218.54327  26538646.18  22583.33177  2860618.862  7372041.152  271818.9094  237541.7431  47286.48328  74009686.07  354907.4156  108255.1389  2827831.399  61549.76475  297461.0401  203620.0969  92532.5672  5431543.814  164952.6596  40383377.92  137773.0997  1311798.054  1839214.054  5322737.673  104480.6564  14847584.68  5671331.379  8334.216936  1840545.696  1897831.053  3717695.227  44015.4972  2068261.343  192636.2898  13915893.43  4945185.529  349915.8043  37231.82306  445654.9007  417245.8674  1807110.385  169941.6986  175793.6223  936944.962  82972.73983  2014977.628  8167112.154  247467.8801  6365979.087  27007.59784  452982.6442  85046.017  48113.33544  1924146.888  1260528.531  169652.7136  112969.3269  14127816.46  307078.2895  95527.89303  82082.94313  2951522.339  22857.57607  78515.29881  1617103.042  7560693.403  574596.6592  152170.3898  4786512.502  101747.1282  298323.8134  17039960.17  7717761.297  2366376.112  530173.0077  70048.16139  427673.2805  41378290.82  105204.7931  3454637.34  50613.47784  14690.85349  436579.2744  384685.354  328207.721  41430.25019  103596.7969  480463.1827  2053970.079  497764.9873  1535354.597  116480.4627  68558.78338  586976.392  184440.2826  4727913.725  37765470.14  33093.68616  29523.16605  121705.7063  1153813.347  41660.81638  128888.9351  633875.2379  9067733.171  2373567.25  46114.91456  22750950.21  15133.26497  1819309.072  718179.7781  5097987.31  374028.6659  53489.99243  491596.6673  1537021.317  340686.8102  10697.32779  19965.77559  1348612.236  16288.52545  56984.00514  88389.76712  3026980.831  21458.65293  805186.4291  36639.03683  35221682.37  449332.5177  1992787.103  5893049.043  4693114.832  81620.76363  7972323.797  3872.685404  243785.0999  16849.74923  19105524.3  15798.93307  6451031.862  2935265.251  571985.6534  910433.308  50212.15704  17707604.78  30025.60548  1148373.924  1866685.575  171294.8699  36917.88778  7403.035089  982141.5529  3120823.288  37029.44324  189093.1039  58885114.54  54585.39749  92556.8167  132916.8034  8246.173764  58926.93548  25872.48764  944339.5573  105558.4691 | 14922951.19  269734.09  142323.6253  1207428.69  48097.19468  4186105.381  1664574.639  187145.9224  3656697.603  817269722.3  54607.39265  12156.74213  60176.58997  4440084.75  13604947.44  50835.71818  2106986.356  87611.42998  2035374.216  338636.8228  51747.8437  2694823.737  20410.22972  103005.5881  65630.39704  192703320.6  774961.9366  629558.7502  184635.7856  15682.05555  747449.1951  469291.04  70764.4096  5373025.002  186474.0455  2081503.637  2283690.119  17075564.61  2981445.955  141197.5916  576493.5863  259352.1532  3033010.262  143243.0382  236422.9326  15707427.86  4483486.316  272836.2125  90721.1847  2327339.919  95468.86301  432227.8356  243878.2294  1548664.659  82425582.58  300474.9668  2104471.182  584024.1282  26862.45291  27689891.03  16315.66961  2571546.687  4974324.02  271632.882  226973.3255  70105.76411  94766336.69  345589.2907  83966.8634  3997604.47  129273.7826  212301.8035  199579.4929  56036.46793  10785542.29  93784.79417  42183504  120437.1304  1290859.509  1573594.082  3988596.363  61427.91358  19462465.83  3294878.936  9507.239757  2015164.358  1848361.472  3748047.069  19760.48969  2595348.312  118763.8347  9246633.855  5517168.813  342893.9653  26313.59234  275671.6938  382201.1245  1999528.429  371838.0089  191178.0351  655474.4857  73419.45132  2174808.983  8515386.012  97446.81217  15566560.65  12751.0984  524702.5717  52931.83594  29497.26369  4199322.344  1890155.145  112590.025  85389.40831  18121207.39  281508.0081  55368.81939  96202.20059  3340851.673  18177.74787  51207.09405  2177336.998  18886554.73  549886.8685  107239.3892  5858633.652  136910.1794  354183.1674  18564253.63  8008393.353  2521329.722  563036.3838  55226.73255  452619.1712  67237214.24  103761.3411  3520288.903  64510.10888  18715.72775  655831.8746  445524.2701  253081.5536  50612.57841  79909.19003  637259.8128  1938502.696  521570.1629  1836821.389  75643.51996  69098.25981  456894.4682  141005.5551  5765392.935  56697168.56  33439.39497  16803.33534  70139.03809  1010433.473  32519.32225  68410.00751  801706.78  11698534.31  1753392.172  52343.48797  26897623.86  16729.226  949669.9549  398613.8546  5317858.238  354109.2642  23806.26115  478195.0661  1211758.582  272578.9932  9020.216796  20529.90303  1677799.635  22679.7293  26224.84492  77967.57707  4042941.909  36264.17294  1033910.992  81288.38276  40787158.33  456915.8772  2315671.611  5611782.493  6285810.869  27282.09582  5666443.704  8695.639578  194709.2355  48124.7989  21442716.22  44406.72593  7434610.894  2396151.261  640481.1229  1188752.454  63959.33305  34239210.46  21958.58435  1305734.395  2001431.159  162411.803  14451.07464  22450.61046  985113.664  4054075.513  56797.10745  147969.0811  58969737.07  60995.45199  78406.52044  202932.3177  58873.03424  86951.95988  37148.29005  1386318.444  141133.4484 | 1793817.975  43128.77017  30477.14255  273937.9134  9831.478452  981089.3252  200921.7878  64490.85737  1950731.316  251541710.3  12934.32657  3071.262387  22335.53792  1908756.48  6935747.742  18333.23597  90063.91683  15813.57944  328606.9507  148839.4461  19753.27481  1449062.916  4701.150862  17057.24172  25788.83728  48401613.68  539513.15  412528.9548  1011.861037  3082.030012  2167782.28  253177.6861  28905.26891  2985509.738  90956.93424  1383512.451  1145295.149  10899290.55  339094.9058  62457.93746  151565.0462  88960.07811  5848504.22  18936.76494  127842.2451  4810624.185  170069.3142  602162.3564  34962.2961  1400076.381  50393.3975  301818.9887  68172.22821  1034547.381  19638075.56  183.2838594  967454.3328  85670.71645  17557.32466  21716799.08  7596.29795  1754788.497  2745713.74  157823.3749  188963.4412  11930.39805  45338265.22  97352.57056  25745.29145  1450901.282  1494.173799  91404.60047  123461.3881  25618.66185  217106.8379  19895.14329  20457862.4  75018.89434  941008.9308  1014318.885  8855630.821  24968.03308  8437646.261  1964490.986  18978.341  1441210.357  1337156.221  2241558.941  5641.919165  5691762.089  91585.1842  5485471.471  2564593.6  277936.9416  10114.58143  120605.3121  242827.8027  3931448.074  62124.9432  55043.7266  392253.3861  41497.49106  3186580.903  4058266.074  28533.29819  299335.8008  4162.570945  1198307.341  15454.71114  71331.89523  420579.9096  1008911.526  43271.52746  156919.116  12676921.78  103874.8377  32796.10675  150892.2082  1465320.573  12955.14237  31201.32284  3073245.086  2024843.704  762601.9366  74352.98825  3281971.717  54984.24094  245243.446  22677319.77  5770984.74  3881928.746  428220.7408  39094.38644  773428.8655  18309803.39  44346.90159  2500204.9  24876.72314  15056.4104  381214.8369  344016.4864  230237.6512  75419.45001  208321.1817  951789.2732  1535793.992  408378.1037  2609895.122  46427.73789  13719.66564  1008956.674  105849.1852  4037835.185  25808115.05  17562.8859  9071.492442  39657.53502  963884.9933  32020.01456  35615.99142  1124033.635  8163121.473  1143451.282  36361.29237  31796178.76  38754.85061  678541.5374  261844.6573  2800036.779  331770.2974  22351.69946  601231.4264  2449497.933  345844.9718  19009.78382  9080.523044  2300287.633  9279.378885  12512.3401  53361.54278  5107792.085  19846.01104  703399.4533  93393.37338  32105753.02  352813.1025  2884768.475  9627976.424  9055870.377  17305.65557  10742535.08  22264.09032  360822.7598  59691.9049  16395062.19  231402.2756  8931739.728  2423075.387  1149751.176  1975612.249  79593.36802  19410495.24  24993.92175  1630325.128  1610049.644  201621.7445  14424.52198  28462.6089  914112.6757  5041434.791  17946.83224  84096.64361  82439303.05  64295.1367  88591.4235  154400.7556  76292.24336  80547.9696  46524.3058  996992.8205  304877.332 | 1652225.065  43851.70257  17531.88255  93821.97907  14271.36123  678657.2681  152083.2134  43192.82871  1402800.829  253762676  13285.68807  1571.957229  19672.10978  1536892.386  7246602.791  12216.80807  51236.58236  14772.85046  369776.7354  100378.9702  17774.42037  1325926.781  4697.576824  13103.73398  22930.6375  42867816.78  486916.1776  256572.4331  102.3046462  515.458206  2250018.268  146155.7105  23600.60087  2323368.273  91129.79062  1351093.935  1207671.212  7237637.789  228523.7049  34659.43375  129683.1698  83103.81627  5120912.013  13628.82012  64160.29938  3737540.649  102176.5086  805622.5201  16603.51158  1051448.201  29197.64437  248508.4418  46019.62652  991484.4335  18350598.48  186.6976903  836098.6823  63181.34955  17861.01575  22503802.52  9689.390082  1011074.176  2229008.4  65652.73961  152801.921  16940.75188  26179210.78  73429.20894  25106.42059  862717.975  1719.069154  79557.02165  130876.0708  25152.12742  209575.396  23254.21978  11578235.62  76059.08345  1035712.236  958163.9664  7808011.651  13713.15285  5357075.249  1801839.147  30467.42969  1191607.393  1190719.478  1619041.587  3774.3164  4221043.235  46538.24526  5437049.496  2154943.671  251838.7389  20237.79535  168488.4338  260875.8916  5326332.722  36705.88698  121209.2045  518768.1916  50550.0565  4089215.073  4767592.855  6835.381612  174421.1295  7479.705486  1940268.995  27775.24661  105499.7032  139123.9285  512696.7263  50412.31363  178609.3245  10067698.15  121728.8595  36453.03749  170272.99  1265060.973  13070.16592  47725.36426  2983110.17  744750.3882  857382.2815  82220.44065  2689706.432  52467.94634  224910.0536  23290546.9  5112099.238  3448945.217  280011.3117  36523.8382  679360.7287  9703149.165  57137.25286  2953646.832  36266.80223  8084.466395  270214.0977  266362.432  111209.4589  101014.2476  261262.5081  865560.3718  1289470.796  228124.4961  2355681.505  58915.18376  20586.83817  923970.8454  104731.7394  3741647.325  14433624.36  18169.5729  6175.685269  38738.70234  550670.0655  28192.98084  50155.74358  1473850.327  6228275.711  1375986.806  27522.84842  34072136.48  44920.36565  738670.945  280878.2843  3435081.975  356633.7599  18054.92482  805822.4553  2161889.735  385548.1396  50096.48953  13178.7123  2470643.699  13467.15923  24199.82922  58201.29361  7542724.148  14392.82464  325204.971  76576.42931  21207248.32  188661.913  2289895.802  13197126.83  6937597.231  14830.38634  12443445.06  12522.4007  493772.6212  57336.27953  13466746.46  116087.9581  8505213.116  2097120.985  836913.2387  1221689.005  59631.35744  7516860.389  9312.382911  1425432.89  1292341.531  238519.0935  14579.24639  29884.44872  876133.6992  3958852.136  14905.12358  113817.6519  74669220.43  78939.41376  113555.5809  63056.07338  74046.39624  104353.959  36672.62144  659720.5579  128592.0522 | 1452355.2  63870.55007  26400.58504  277784.2188  11771.28648  808829.2633  150034.4698  41493.10918  1781904.097  316288201.9  13031.37333  1056.74998  21683.35398  1858815.444  7596218  14773.57422  56570.16734  16712.37701  350294.3387  136516.3896  21946.22771  1323341.685  3092.003007  13565.77695  25431.41864  51136015.29  552592.4597  306318.4338  200.6137047  3579.411299  2045297.348  204147.3443  39012.68425  3171373.885  104566.2924  1411268.819  1544526.828  10400097.23  366893.2504  46327.39685  192366.3303  132371.6165  6608754.446  26362.88908  86106.69805  5828813.855  136999.7445  879009.3799  19270.44774  1065423.461  37343.9424  286090.1175  31772.23187  1056834.984  26577893.35  311.4934988  1092241.278  102985.8529  13771.42464  23914007.01  8799.190729  1461409.395  2412320.097  85762.48771  173201.528  20621.36376  36714907.94  123616.1878  21170.14295  1024235.701  2671.565275  105319.4379  151955.4323  17332.99163  139413.9561  35569.21328  18169352.95  93967.02372  947442.85  1173192.137  9957216.616  21117.68792  7715249.695  1750846.098  23172.58506  1302158.933  1276457.908  2367994.255  7160.558265  5324063.472  61592.42842  6143895.463  2763365.016  307277.3184  16471.89212  148321.8679  323097.3164  5429503.856  42558.18337  125537.2988  552944.7751  53486.21799  4295352.532  5648010.367  31792.16533  157528.7701  5435.84864  1739735.812  33328.5043  110310.3801  150480.3924  631880.5126  64553.6949  216645.9131  11282801.07  203306.0495  37637.8472  200283.5333  1980864.933  15226.70665  39689.32321  3744166.442  783226.3789  825127.358  98254.04981  3929871.908  43575.52485  238684.7643  26785926.42  6550974.875  5323903.96  237793.9104  33914.15019  732934.6989  10496810.23  63305.91917  3128565.016  31236.80512  7485.448952  265427.0222  317208.5436  164712.7808  101427.5071  435538.1048  925066.3139  1669874.945  260247.1399  3661685.178  56121.27931  22482.21298  1242442.272  59524.54133  4614106.817  19681251.33  23165.22439  5674.841839  46915.63154  531092.227  28690.85809  45677.15589  1665700.977  7140754.355  1521873.701  36544.22227  28925622.96  34833.78159  938540.1997  365130.4005  4223134.645  315950.5903  21026.34996  907377.0926  2534306.571  368716.8542  47705.46313  14982.35146  3025263.56  13401.77489  13878.78613  75569.12944  7175483.838  15513.10575  340738.3816  84369.99145  26340186.18  349404.5738  3043454.702  10304085.86  10397420.26  20747.02475  12239185.62  13021.11276  504720.0912  63606.47216  15414431.69  78158.75376  10124597.97  2554309.761  1553386.86  1602971.724  66758.05659  9044253.552  15317.71715  1579123.841  1763891.231  395739.3906  16756.97463  39068.85768  930761.0777  5222406.137  27467.48772  87062.54112  83849657.03  82605.34679  141184.1517  82358.58982  73731.00821  129744.3105  51917.0093  833679.7329  250478.0598 | 1304339.283  48433.99944  24582.7148  187654.0758  13807.01972  730310.1081  93115.88801  39732.19009  1368982.533  268198751.6  14905.68452  2490.527656  18397.77493  1449672.915  6469275.456  16400.7348  49527.26201  7282.428987  286988.408  137609.4577  16493.54127  970762.7707  4174.126243  12382.97041  21579.72331  42241832.3  445840.311  313175.1674  584.796011  4070.767781  2054065.14  135892.0143  18204.07226  2401015.51  67540.84752  1288794.986  1080413.736  7451676.005  299032.9747  38071.78259  137913.751  110583.964  5736924.153  22541.83913  77856.37407  3518118.765  100805.8491  877350.559  24256.79579  891935.1344  39661.4126  244824.7552  36920.40076  876910.4251  18112321.97  492.1954317  800275.2821  84720.4339  20045.19088  20915082.22  4868.284206  1126583.254  2283235.377  67170.68271  147280.0359  18449.96485  27208102.89  63202.33455  19700.48816  807241.5325  893.5080767  63964.21219  94961.23181  19719.63473  185396.2173  29329.21786  13824486.63  80960.90086  738925.4804  762304.9686  7530903.561  22229.34093  4956504.371  1950566.424  20050.13993  1115318.538  1388129.061  1691395.314  4962.444498  4910765.507  60453.93536  5181582.286  1941501.867  258457.1478  14778.22622  138574.3137  266961.0267  3609026.362  30310.98627  105922.492  425989.942  27413.65001  3463285.864  4462442.077  27055.73416  164940.2423  6982.040793  1184813.757  20189.61781  99823.17376  212012.5464  608627.7208  70852.4364  194221.9196  10125924.05  124430.0828  36037.23681  141443.4479  1309935.899  9415.415704  31528.7218  3329111.24  936457.4603  699082.5146  72839.9353  2987912.583  47128.97857  189551.1759  20083017.04  4414403.961  3838381.115  266053.302  41503.84511  667603.7996  11910655.65  31532.12098  2821873.69  33432.23986  8447.366737  256279.0609  287820.7404  159634.9632  74617.25624  315151.4891  754423.0532  1372440.864  239663.466  2467332.681  24061.2842  34465.35448  781044.955  113046.1239  3593819.985  18128767.52  24964.87847  10760.21199  27178.03841  593927.4653  21399.75153  59062.15083  1131930.821  5933123.25  1419951.938  17971.59239  27395673.83  17674.04338  792024.8349  263661.7044  3325961.081  296131.9391  14749.54209  852837.3767  1866431.56  355864.5042  40503.36804  7896.878302  1917632.283  7652.11734  4214.787893  69581.86583  4843737.002  7263.328301  474495.9131  81083.02911  22236090.61  274214.8457  2464488.498  8421299.438  7109099.765  25206.6159  9860357.848  9694.431746  299583.9869  45926.1619  12212450.69  96567.1255  7190506.526  1989604.564  845933.6293  1480970.42  72380.63826  8584877.408  11547.67869  1440202.646  1360015.343  277762.7896  14129.49995  20040.29046  813946.2155  4335067.384  11363.39783  50557.03089  59554988.64  66051.3767  115959.9504  83083.97919  51453.69431  109132.2856  38834.77859  604976.7809  207644.829 | 1869666.303  59420.43688  27800.64935  241145.3488  11714.74033  1015273.78  194376.2199  59280.4797  1613017.689  335756874.3  6136.226689  2592.134084  20873.82779  1745172.353  6084570.025  17825.98602  94517.66279  26937.97754  329722.1721  115647.0617  24072.37628  1260564.317  4187.752285  24904.44053  26672.33687  51376731.97  485259.7253  337399.5303  406.6669824  983.6467745  2495721.176  208006.7823  27150.1093  2415860.629  87892.12604  1339634.547  1042390.608  8485445.92  472463.1002  69051.8646  114107.4917  73511.33172  6499676.155  24637.12113  134462.7543  5097740.336  196421.6263  905914.9686  38222.68709  1467218.784  45885.36075  258464.2047  89769.99008  1073808.609  31184927.7  179.0446278  920468.3226  97056.51613  16881.47453  22505525.9  7113.45137  1540125.082  2426018.569  117666.0969  159941.0678  26707.22216  40789856.44  147153.2097  33656.7004  1220414.098  2076.013303  78146.90544  117538.6038  20949.83923  283798.8137  24263.35303  19616724.97  95072.31924  979821.3785  799153.7377  9625834.005  30244.66736  8755802.17  1699990.028  26650.8772  1583499.906  1499964.461  2205258.203  6177.964884  6638758.609  66609.52378  4276289.139  2672281.27  231014.9165  11835.93914  108042.3076  222014.1116  5967459.084  54007.7994  116376.119  426117.4163  33262.60217  3401200.679  6060291.404  21389.78052  362254.7748  1846.105092  1936390.845  39724.15207  117061.7129  565527.7316  690312.544  43915.61228  249156.1903  11543854.73  203640.8153  22204.46282  223847.0574  1987011.301  15584.40958  30398.82875  4450094.973  2595154.552  798483.3708  52788.35813  3386910.779  67322.96737  243644.3473  24312048.6  6541885.318  4618550.827  453348.8386  28429.04587  961793.978  27176635.17  88788.03582  2313153.684  48874.7164  8599.121366  294381.9757  367570.7369  202907.0323  131355.9249  444964.5877  816363.0839  1328702.808  430473.7856  3140597.817  7094.483006  45942.03931  912410.2681  120823.8626  3991276.855  30769697.66  19903.27979  4966.753924  29229.91922  771880.8131  20637.507  34827.81886  2003470.169  6880546.88  1187397.708  34199.35666  33199015.55  32346.28895  608765.502  251997.509  4253062.188  309932.9689  21193.40861  1155777.862  2313777.707  361844.1064  68931.22549  19223.33473  3487230.132  11818.18627  13369.6718  65997.61978  8498465.412  12279.08577  594080.6451  103249.9454  28748879.76  247994.1238  3017066.426  7936021.385  12732987  15863.97945  8947822.537  22436.60095  549981.8094  41134.14658  18030464.37  175466.3157  7964682.163  2097208.969  981217.5786  2012676.881  76746.19647  14392810.96  20512.58102  1392159.033  1643167.128  359013.3608  6226.637594  20522.08965  923810.6465  5356091.974  22582.73283  91683.19438  66904164.43  89839.66147  109434.2955  117363.8484  114574.8028  128312.2597  68927.26681  758466.6022  236043.0985 | 4.18E-09  6.53E-08  2.60E-07  8.67E-07  1.56E-06  5.85E-06  6.55E-06  9.58E-06  1.07E-05  1.70E-05  2.07E-05  2.09E-05  2.24E-05  2.34E-05  2.74E-05  3.23E-05  3.95E-05  4.02E-05  4.39E-05  5.03E-05  5.14E-05  5.55E-05  5.83E-05  6.41E-05  6.50E-05  6.65E-05  7.84E-05  8.92E-05  8.96E-05  9.85E-05  0.00010449  0.00012662  0.00012708  0.00014452  0.00016591  0.00017349  0.00017898  0.00019765  0.00020382  0.00020722  0.00027478  0.00029301  0.00029581  0.0003133  0.00032439  0.00033381  0.00033462  0.00035976  0.0003643  0.00037172  0.00037403  0.00037501  0.00041024  0.00046029  0.00046273  0.00048183  0.00049138  0.00050869  0.00051697  0.00051886  0.00052938  0.00053248  0.0005686  0.00061801  0.00066557  0.00067521  0.00075538  0.00079702  0.0008272  0.00096584  0.0010319  0.0010466  0.0010961  0.0011512  0.0012943  0.0012985  0.0013024  0.0013633  0.0013669  0.0013994  0.0014193  0.001456  0.0015899  0.0017305  0.0019656  0.0020053  0.0020064  0.0020388  0.0021243  0.0021889  0.0022417  0.0022954  0.0028996  0.0030654  0.0031196  0.0031522  0.0031616  0.0031717  0.0032215  0.0033272  0.0034044  0.003416  0.0034178  0.0034322  0.003667  0.0037163  0.0038148  0.0039539  0.0040329  0.0041724  0.004512  0.0045623  0.0047013  0.0047248  0.0048431  0.0049136  0.0061989  0.0063616  0.0064013  0.0064452  0.0065698  0.0067077  0.0070462  0.007233  0.007434  0.0080786  0.0080825  0.0085476  0.0085751  0.0091661  0.0093128  0.0093876  0.0094349  0.0096442  0.0096776  0.0098212  0.0099007  0.010106  0.010107  0.010217  0.010507  0.010643  0.010809  0.011082  0.011464  0.011665  0.011876  0.011888  0.012014  0.012101  0.01256  0.012771  0.013581  0.014119  0.014463  0.014538  0.014655  0.014701  0.015298  0.01545  0.015644  0.016243  0.016244  0.016513  0.016891  0.017955  0.018305  0.018791  0.020271  0.021054  0.021995  0.022136  0.022337  0.022963  0.023509  0.023571  0.023634  0.024534  0.025456  0.025493  0.027312  0.027612  0.030237  0.030988  0.031684  0.032284  0.032822  0.033034  0.03376  0.033887  0.034265  0.034562  0.034935  0.035032  0.035295  0.035376  0.035399  0.035633  0.035804  0.036631  0.037078  0.037601  0.037708  0.037873  0.037973  0.038439  0.03899  0.03985  0.039863  0.040156  0.042372  0.042493  0.042523  0.042625  0.043343  0.043869  0.044743  0.045018  0.045992  0.048087  0.04974 | 1.667224579  1.663729985  1.664953943  1.660880549  1.65225903  1.645970541  1.646814935  1.6438244  1.648149584  1.63889729  1.633944778  1.634786427  1.633186747  1.642407239  1.632608088  1.632488  1.630327781  1.627881319  1.623928122  1.631424763  1.621719128  1.631752206  1.617434412  1.618601929  1.624416765  1.615934836  1.623680351  1.621388848  1.617227972  1.612599946  1.61515337  1.616254136  1.613273845  1.60957012  1.600940561  1.597741658  1.600725259  1.610534587  1.601844524  1.600685345  1.587038474  1.584518483  1.582789472  1.581656087  1.588347162  1.59159959  1.586166684  1.592501667  1.58194902  1.587763911  1.579628787  1.584109592  1.57517546  1.580629899  1.577015066  1.5745385  1.572162107  1.576709054  1.559489302  1.57518824  1.566243787  1.579742696  1.565505642  1.57631824  1.576109719  1.5572833  1.56957857  1.558966062  1.551818913  1.55799341  1.549379071  1.545811198  1.549851522  1.535066744  1.539478332  1.528255618  1.544476012  1.5270598  1.532316219  1.532402407  1.522345573  1.521443937  1.536427478  1.512748069  1.521725013  1.517952929  1.509968745  1.523782053  1.502653113  1.500117019  1.506404639  1.502792358  1.50102802  1.495940876  1.473568653  1.478325979  1.487114422  1.487237461  1.49288925  1.466462414  1.471899183  1.48690814  1.482511968  1.477288197  1.469755212  1.481437814  1.461237832  1.476875662  1.457692719  1.482592979  1.468774552  1.477194263  1.45071587  1.467899169  1.473122059  1.451234427  1.431098358  1.434452285  1.442027238  1.434848107  1.421418037  1.431680322  1.434556608  1.425119977  1.417645845  1.428550028  1.419544869  1.411970354  1.395878378  1.407128173  1.392539272  1.403454929  1.398870948  1.395931426  1.405283124  1.392175613  1.392560379  1.38067767  1.406898114  1.409699082  1.402103327  1.397781549  1.392132176  1.3913082  1.36195335  1.395683191  1.386870135  1.365567062  1.3803702  1.362616452  1.357413978  1.354098059  1.381112519  1.370957683  1.346140027  1.351970438  1.348221461  1.367339889  1.360741641  1.333951053  1.351396519  1.366282982  1.334075008  1.363732404  1.333188747  1.316694947  1.320670772  1.321623395  1.311199927  1.308560154  1.301964057  1.321666335  1.292746895  1.302802885  1.316998042  1.288502624  1.292518856  1.298701442  1.280305391  1.27447155  1.282977082  1.299804225  1.278681291  1.243439832  1.280491903  1.277423345  1.227826962  1.250171694  1.23449217  1.234831209  1.243348937  1.222289856  1.245806958  1.216247519  1.256377762  1.220093352  1.212567193  1.245144284  1.212188572  1.214353607  1.212826542  1.250807659  1.237330796  1.201855685  1.250006248  1.229927497  1.216826362  1.196892218  1.240468651  1.198475286  1.225695695  1.211106184  1.179072265  1.215702374  1.21476125  1.227348507  1.192309979  1.207931188  1.185752966  1.217835734  1.153297301 |

**SUPPLEMENT TABLE 2B** Lipid Metabolomic characteristics of prefrontal cortex between aged marmosets and mice in sevoflurane group.

VIP, variable importance in projection scores.

|  | **Marmoset** | **Marmoset** | **Marmoset** | **Mice** | **Mice** | **Mice** | **Mice** | **Mice** | **raw.pval** | **VIP** |
| --- | --- | --- | --- | --- | --- | --- | --- | --- | --- | --- |
| PC(18:1/22:5)+AcO  SM(20:0)  PE(18:0/22:5)  PC(16:0/14:0)+AcO  SM(20:1)  DAG(16:0/22:5)  PE(18:0/16:1)  SM(14:0)  PC(16:1/18:1)+AcO  PE(16:0/22:5)  PA(20:0/22:5)  PC(18:0/18:3)+AcO  SM(18:0)  PE(18:2/22:5)  DAG(18:1/22:5)  PC(18:1/18:2)+AcO  PC(18:2/16:1)+AcO  PE(18:1/22:5)  PE(14:0/22:4)  LPE(22:5)  PI(16:0/22:4)  PC(14:0/14:0)+AcO  PC(16:0/16:1)+AcO  PE(14:0/22:5)  PE(P-18:0/16:0)  PG(14:0/18:1)  PE(P-18:0/16:1)  PA(20:0/22:4)  SM(16:0)  PC(16:0/18:2)+AcO  PC(18:0/16:1)+AcO  CER(14:0)  PC(16:0/18:3)+AcO  PI(16:0/18:2)  PG(18:0/18:2)  PI(18:1/18:2)  PI(18:0/22:4)  PI(18:1/22:4)  PE(O-18:0/22:4)  PE(O-18:0/16:1)  PC(16:1/18:2)+AcO  PE(16:0/16:1)  PE(O-18:0/16:0)  PE(P-18:0/22:4)  TAG56:6-FA22:6  PE(O-18:0/18:2)  PG(18:0/18:1)  PC(18:0/22:4)+AcO  PC(18:1/18:3)+AcO  TAG52:4-FA20:4  PE(18:0/22:4)  PC(16:0/22:5)+AcO  PE(P-16:0/20:4)  PE(18:0/18:3)  PE(18:1/22:4)  PC(18:1/18:1)+AcO  TAG54:6-FA22:6  PC(18:1/16:1)+AcO  PC(18:0/18:2)+AcO  PC(20:0/18:1)+AcO  PE(O-16:0/18:2)  LPC(14:0)+AcO  PG(16:0/22:4)  LPC(20:0)+AcO  PG(18:0/20:2)  DAG(16:0/18:0)  PE(P-18:0/18:3)  PG(18:0/22:4)  PE(18:0/16:0)  PE(16:0/14:0)  PE(14:0/22:6)  PC(14:0/18:1)+AcO  PE(18:0/14:0)  TAG56:5-FA22:4  PG(16:0/20:4)  DCER(26:1)  PA(18:1/18:2)  PC(14:0/18:2)+AcO  PC(18:0/14:0)+AcO  PG(18:2/18:2)  LCER(24:1)  LCER(26:1)  PE(14:0/18:1)  PE(O-16:0/16:1)  PI(18:0/22:5)  PI(16:0/18:1)  PE(O-16:0/18:3)  PC(18:1/22:4)+AcO  PG(16:0/18:2)  PE(18:1/20:5)  HCER(22:0)  PE(14:0/20:4)  PE(O-16:0/22:4)  PE(18:0/18:0)  PC(20:0/22:4)+AcO  PS(20:0/20:1)  PG(18:1/18:2)  LPC(16:0)+AcO  PE(16:0/18:3)  DAG(16:0/20:5)  TAG56:6-FA18:0  LCER(24:0)  PC(18:2/18:2)+AcO  TAG58:10-FA22:6  DAG(18:0/18:1)  PA(20:0/18:1)  PE(P-18:1/16:0)  TAG56:7-FA22:6  PE(18:0/20:2)  PE(16:0/22:4)  LCER(26:0)  TAG54:4-FA20:4  DAG(16:0/16:0)  PI(18:0/20:1)  TAG54:6-FA16:0  LPI(16:0)  PE(P-16:0/20:5)  PE(P-16:0/22:6)  PE(P-18:0/18:1)  PC(18:2/20:1)+AcO  PI(16:0/16:1)  TAG56:4-FA20:4  PG(18:1/16:1)  PE(16:0/20:5)  PI(18:1/20:2)  PG(16:0/18:1)  PE(18:0/20:3)  PE(O-18:0/20:4)  PE(P-16:0/22:5)  PG(16:0/18:0)  PE(O-16:0/20:4)  LPC(20:4)+AcO  PE(O-18:0/22:6)  PC(16:0/22:4)+AcO  LPC(18:1)+AcO  PC(18:0/20:3)+AcO  PG(18:1/22:4)  MAG 22:0  PE(18:0/18:2)  LCER(22:0)  TAG56:8-FA20:4  TAG54:7-FA22:6  PC(20:0/16:1)+AcO  PI(18:0/20:3)  PG(18:1/22:6)  LPS(22:4)  PE(P-16:0/16:0)  PI(18:0/18:2)  LPE(22:4)  SM(22:1)  PG(18:0/16:1)  PC(18:2/20:4)+AcO  PA(20:0/16:1)  PG(18:1/18:1)  PC(16:0/22:6)+AcO  LPE(16:1)  PE(P-16:0/18:1)  PI(16:0/20:2)  LPI(18:1)  HCER(24:1)  PC(16:0/18:0)+AcO  PG(18:0/18:0)  SM(26:1)  LCER(20:0)  DAG(16:0/18:1)  HCER(22:1)  DAG(14:0/22:6)  TAG56:5-FA20:4  PE(16:0/20:2)  PC(20:0/20:4)+AcO  PC(18:1/20:2)+AcO  TAG56:7-FA16:0  CER(20:1)  PE(16:0/18:2)  PI(18:1/18:1)  LPC(22:6)+AcO  DCER(20:0)  PA(20:0/20:1)  HCER(26:1)  HCER(24:0)  PE(P-18:1/20:4)  PE(P-18:0/20:1)  LPE(20:2)  PI(16:0/16:0)  PE(P-18:1/18:3)  LCER(16:0)  SM(26:0)  PE(18:1/20:2)  PG(16:0/22:6)  DAG(18:1/18:1)  TAG55:5-FA18:1  LCER(18:1)  HCER(26:0)  PE(O-18:0/20:1)  TAG58:10-FA20:4  PI(16:0/20:4)  LPC(18:0)+AcO  DCER(22:0)  TAG56:6-FA16:0  TAG56:7-FA22:5  PG(18:0/20:0)  FFA(14:0)  PE(P-16:0/22:4)  HCER(20:0)  PE(P-18:1/22:4)  PG(18:1/20:4)  DAG(18:1/22:4)  PC(16:0/20:4)+AcO  PE(18:1/18:3)  SM(24:0)  DAG(16:0/22:6)  PC(18:0/22:6)+AcO  PE(16:0/22:6)  PE(P-18:1/18:0)  PC(16:0/18:1)+AcO  LCER(14:0)  LPI(20:4)  LPI(18:0)  PE(P-18:0/20:4)  PE(O-16:0/20:2)  FFA(22:4)  HCER(20:1)  LPE(18:2)  PE(P-16:0/20:3)  SM(18:1)  LPE(16:0)  PI(18:0/20:0)  PI(18:1/20:3)  FFA(14:1)  DCER(18:0)  TAG52:2-FA16:1  PE(18:1/20:3)  TAG56:7-FA18:1  PI(20:0/22:6)  LCER(18:0)  LPE(20:0)  PE(16:0/16:0)  DAG(18:1/18:2)  PA(20:0/20:4)  CE(22:6)  PG(18:1/20:2)  HCER(16:0)  DAG(16:0/20:4)  PE(O-18:0/18:1) | 383423.9179  299208663.6  24073929.02  6294135.234  5698661.136  405930.2951  661038.8385  2084715.383  4295310.275  3570455.511  2193833.81  147135.0966  800314240.6  101098.2066  154032.5864  2345902.494  254219.3692  2088692.076  23026.08037  199931.3637  259285.4043  112782.9948  18609138.46  20080.31881  15928506.95  52776.30257  233459.9242  13702630.38  69125073.64  10844376.54  2650869.39  437913.0368  726876.3501  119280.8882  59284.1337  68533.61498  500005.5201  74381.7476  12582493.6  247290.194  340303.2381  824050.2799  3103281.071  63333919.62  52802.56672  133966.8553  2108293.57  2133052.36  129763.078  88548.35399  31140468.15  2715515.609  5403427.673  129175.4952  2434560.437  20947998.48  161180.9589  4449365.3  4620336.447  279280.0447  111101.499  23044.20654  47548.85004  5976.653459  44159.93599  1105844.137  59289.50262  203988.4463  2856535.401  126573.7514  23248.03578  2281230.856  24002.77819  60699.96941  586729.6714  40102.69492  139773.031  88657.57082  435614.8835  41316.06265  2360129.736  126209.8403  141546.7348  144822.2335  186265.4733  1886968.535  14168.85988  270192.5308  55049.64675  17262.23631  1129657.348  15123.92653  4440757.33  933055.4047  53166.91027  6369.810379  73665.39598  2143623.108  56067.78633  2153.693054  94238.46777  929739.5737  282521.8656  18438.67454  631612.1232  4961049.764  17202045.62  335843.0297  1185649.207  4102507.337  64751.69839  161804.4991  1876958.685  33911.12505  100771.6234  8582.725585  8783.899363  5806546.559  18981720.67  55659.2155  51804.80597  48939.10529  247291.5158  3598.983172  17741.83154  9424136.55  4332433.168  3272439.937  529419.0948  347598.5048  2246731.325  51973.97348  2840925.015  1761114.11  710374.6813  1582401.602  50625.20777  4173.981483  3394800.844  39923.91593  47803.38888  46135.09896  19430.08231  1380113.221  13856.71009  8861.311825  3016549.545  171677.8841  1861957.962  1384618.907  28417.11399  455496.3796  460623.7778  1792576.009  5937497.479  126764.415  4714463.81  44885.49285  8044.875909  14545071.72  3913620.306  86551.17361  2485836.374  115195.3228  2589781.592  224549.6212  2934.715737  102779.7386  324052.9652  47535.04415  239534.8017  88810.77151  5239448.036  1262960.362  495319.1665  8418.014605  256903.6288  1276670.725  3701426.71  6259651.945  17621229.14  5061332.129  82177.09778  825619.6915  30606.12207  78063.70396  1628839.843  355123.5748  30359.711  1383022.61  47882.10911  24793.67838  757640.2382  654542.7652  40385.71123  2416323.093  1441732.747  40394.5533  58902.03482  29003.41133  163486.5772  3382726.378  14675198.26  514860.9066  13374759.89  170193.9687  107774.5701  19898974.68  68806.75493  20160705.09  746564.115  3329540.597  7428519.468  100874.123  139701779.9  14809.20488  17466.1754  107915.9726  30662779.48  105112.0343  4721909.859  22814.76525  75769.56757  418757.2303  36868978.79  602686.0734  1224065.39  167130.5901  118681.5737  8311438.201  258171.6219  842107.5544  105048.1453  27462.97095  1417222.412  10453.28835  3099221.759  438418.7729  8787475.541  159409.7664  21556.63784  174062.4623  2081495.869  3034555.206 | 409177.3838  307679498.6  25412806.25  6514709.477  4896450.093  358968.3421  655724.1273  1905762.185  3694063.197  3981349.585  2810049.513  117694.2284  705296242.5  101093.3513  130170.2144  2253823.334  212207.5523  2120159.431  30231.58589  141581.637  319822.8155  149351.1786  16141436.8  26421.3424  15510756.1  41093.57202  223989.8253  16033907.43  52137622.31  11138839.36  2387207.985  303873.9624  557194.3685  132603.7144  63750.64501  68353.87778  668974.6661  103217.8331  10480455.66  211270.367  241455.6467  810449.3038  2482841.8  76954091.13  41005.77369  133813.0142  2628196.3  2573722.325  91167.91963  104228.5288  34937610.07  3197612.915  4315056.404  82169.21926  3046306.82  19037967.57  170632.7767  3188292.025  5984317.803  297577.8299  93043.41479  27752.79511  54211.94059  4973.529  53234.00771  1046840.366  46380.92982  334372.9686  2475896.005  118919.3972  23629.89961  2281477.749  29260.51552  50228.85015  477525.9128  91857.12867  154584.5718  109787.3026  434674.4266  25650.48624  5613029.033  296194.1291  229587.6178  84632.87656  379030.9336  2687717.175  6968.788349  246667.3129  62320.50922  15129.49639  5754040.939  20539.23449  3306783.303  729928.8042  37719.17729  10604.54218  98765.08337  2099747.936  51308.40005  2628.141857  92947.76269  2618376.05  228893.8497  45316.41919  1353613.167  6351183.502  17084378.84  317256.6067  1814371.54  5117571.193  211940.3467  136012.4221  1682653.937  42679.4555  88851.49155  17509.35368  3641.558058  5154387.531  24118206.34  60987.44936  90468.8077  46694.75012  174676.4116  9924.46954  25954.57271  8872226.13  5493184.568  2436163.987  614771.814  502239.1492  1501886.025  45461.13269  2497288.661  1769391.312  771401.8935  1857879.453  48365.17261  5312.774823  5182640.251  84522.46815  51249.27429  33100.80807  20644.59457  1589825.142  14042.2708  5919.366523  2508767.217  274598.446  1161629.369  1843413.793  36347.8617  378894.9902  493206.0543  1251740.601  7352543.379  78305.56063  8932717.839  51883.01298  11518.37311  70336428.59  3564239.109  119939.6097  11460238.18  346638.4207  3017680.922  970007.2156  3051.323623  110703.4553  424141.1401  59211.3729  210880.2733  67005.86678  3676339.822  1233225.868  758637.9296  11502.27083  144258.8866  3393653.289  18289273.69  32964236.55  15815260.19  13529809.58  115109.57  1542143.455  45249.61872  163059.1638  9242759.132  515619.5617  33147.01889  1244946.222  56903.59051  98781.63326  4522845.141  1052945.66  29312.33915  2692517.55  1390593.204  40645.87245  68187.62967  31464.44211  162293.7024  3302324.965  14980964.82  2043308.833  28520122.07  171481.4128  118095.8281  17462536.69  57041.59585  56041706.36  646720.7999  3536053.597  7592216.16  123942.7601  125017749  22836.99404  21186.70638  167746.8022  27171891.94  164537.0856  7264897.034  40608.90496  57955.68874  717944.2462  32452419.86  613096.5208  1149251.334  189500.195  119273.7033  5003975.34  164207.5464  1088393.356  90276.288  25317.46678  3587638.576  16206.69076  2580080.193  266549.4554  7350835.398  158559.261  22794.86916  329812.6235  1921517.848  2566061.62 | 379033.6055  341581964.9  21765186.61  6061670.023  5882799.29  403706.9662  644834.2804  2412829.79  4214278.948  3196192.909  2239874.466  139168.1938  797469673.2  78005.70576  173234.6354  2262345.985  221326.6808  1558193.999  32308.99525  191138.8789  279590.3108  140448.834  16717245.07  18246.85857  14187338.56  55241.43631  261861.813  17493413.08  61179027.46  11467382.23  2892299.268  416081.6162  534023.3763  127388.4416  62096.71538  78258.63812  712847.9287  106350.6614  14984786.63  285076.819  272283.2087  929205.2644  2756180.08  82656939.1  34671.26463  154011.4311  2469476.274  2751379.82  89644.03999  123158.6486  43455972.85  2210310.211  4104368.55  135268.1542  3565297.599  21717274.88  253316.8605  3647833.205  6188428.865  380728.8506  112729.8053  30486.33691  48344.05668  6574.717895  47627.67711  1179463.698  47131.6995  345082.192  2452505.859  72515.05847  19513.93306  2391937.019  29186.64884  72728.06014  457297.6574  77133.69274  120640.8501  125240.1106  485208.5709  37338.36184  4557517.475  301766.4368  286530.9503  126786.7849  255199.4855  2323933.768  14130.04039  351583.6036  77498.22228  13638.84105  4864182.999  26104.91881  5216978.637  744938.7443  60096.44678  16248.16726  117045.4337  2379107.902  36687.20088  7450.792518  67098.60274  2201361.444  254596.6961  42452.39876  2433453.901  5900137.931  17142034.63  452417.9529  1647977.383  5718844.033  197724.1158  134915.968  2073278.772  43026.46641  142727.6493  15462.09502  6823.223767  4821709.47  21465876.57  55016.67068  69251.69145  54381.60666  188062.5417  9252.577441  27380.75336  9331118.613  5508762.17  3493774.843  573622.6462  456690.8732  2083892.419  51922.69231  3015643.717  2264363.613  861769.6556  1910811.762  41220.8616  5635.29497  4221638.475  87948.86383  40694.54367  55337.71607  26663.64356  1747850.273  18101.48229  11983.14376  2517292.46  249645.434  2499750.201  1931064.913  48290.90067  405664.977  591166.5338  1437364.043  5673931.624  124650.7265  9439312.768  50992.06637  13906.83868  56427423.03  3737781.418  133825.5395  13429262.18  402407.5857  4714783.472  802529.7722  5505.590977  93060.66088  394958.9388  63098.92581  230923.2646  74369.59095  4584579.729  1331762.982  666181.6524  8649.912189  249783.157  2609038.092  16424707.38  28606919.41  15988203.68  10229662.05  140984.2898  991464.3727  52835.18957  147186.3493  11573082.36  558327.4145  30781.19541  1771600.12  60432.45496  63446.05892  4483693.851  1008777.426  49116.08578  2601138.145  1749738.927  33134.62314  106966.2377  42017.18455  166919.9212  3169390.351  16819880.38  1719271.024  27598551.56  195266.8542  212034.9995  18047891.97  61139.47391  62162802.02  789427.2186  3221844.908  7253808.692  107075.5058  138744030.3  22015.1753  25376.57834  192781.0056  32879919.3  191656.4107  6977710.908  58504.65743  85548.05071  724172.5733  43422295.29  765161.3039  1242048.372  191022.4203  65435.51568  7059919.301  230356.362  1120080.357  94837.95088  19022.81889  3516124.937  18672.12858  2735065.497  311366.9723  8843791.473  87043.38815  15882.83387  313461.1359  3516040.421  2886299.694 | 62515.57478  51414464.98  2210535.076  2415157.998  1023651.127  85914.98948  304852.396  305973.2552  1466201.683  601525.1684  189271.381  28798.82622  347224195.1  10419.50272  24626.65165  778468.7177  71718.15876  216251.7844  519.3558758  19084.31559  103334.2529  13883.86155  7586179.482  528.8705297  8541445.906  5732.720601  74552.69988  6118668.665  21975376.38  5368948.472  1193085.295  71062.67826  142148.6873  48954.8606  23705.91452  19731.76053  126503.3491  20220.25816  3638619.774  81785.90165  99622.28355  422366.6076  1447539.613  29713466.48  99766.8897  49822.48835  1101222.857  1417787.303  23131.73416  192125.4061  14672375.98  1034851.022  8854323.836  20466.56665  879596.0492  14361564.21  813125.607  1452285.427  2174099.215  997351.5622  34348.84001  11556.8581  26877.50066  10728.65974  29888.31081  1462223.418  13568.91974  66180.23511  1834075.493  23870.41615  6085.669986  1019315.65  10398.83993  19602.17583  888367.8222  2067.120483  68741.44478  57757.18317  277300.0997  13577.55063  65020.00175  312.506639  44161.98078  34744.5768  31303.28596  1084651.334  1546.867038  106091.1681  27210.46287  38374.29245  16350997.93  7996.21302  2074927.363  577360.1423  26412.26102  77432.77444  31060.73119  4978271.929  28964.28271  17692.10224  165798.9121  46084.44551  146122.4419  115761.1984  3734689.362  10665307.37  11161398.62  853385.0915  659982.2686  3038370.564  454.0426917  306452.3111  3298449.926  99760.14806  281489.349  50950.35402  13516.73762  8539557.425  35353871.14  34237.29962  24013.48533  130800.2572  108125.8751  15798.58979  7685.061676  5627378.218  2975884.112  1797261.7  867126.2848  237223.0185  1059143.134  93786.0304  1971286.797  1489769.395  1370840.773  1192411.572  29689.04134  10957.84981  1873134.888  11942.811  72877.92739  83718.94723  45013.82486  886513.2756  30383.27364  4181.932738  2122137.396  87055.15148  953147.8306  3150006.382  14433.23297  307414.5175  241021.0401  965838.6916  10513800.77  70766.98703  14071105.91  18682.30454  28512.81773  80290227.53  3414851.558  46464.6864  204087.4013  41323.52188  6404375.34  2943940.594  9525.940349  180688.6401  277588.249  82420.43604  170699.2345  122275.7887  3312658.135  834807.5121  247073.3921  20648.78417  121333.1658  3896727.027  923553.1271  36437236.35  9868432.48  15533071.18  63611.38194  516744.6403  7045.368513  328485.9913  304851.8181  274296.1601  42042.89234  2448408.125  28829.94284  86580.8603  263538.3324  1287516.157  102404.9534  3554604.88  2497936.871  58862.73681  111253.8286  15680.2673  136734.4782  2786028.121  12166066.98  2478488.46  8214886.146  237109.5407  45712.32378  24047082.31  26783.00566  16457835.53  1887042.544  3795809.372  9973277.928  250817.6624  126119462.3  29266.55241  36336.17184  348119.1571  24114316.94  67832.24245  3416030.975  67774.9301  59627.81446  790917.4121  32946843.77  2073787.913  1440311.591  109257.5625  61265.66964  4727930.586  165384.0393  663723.0973  168303.2533  41498.38965  3002610.344  35733.2284  2612286.121  241388.5137  6005073.948  171201.5375  9874.489198  364820.0563  4974597.492  3238985.183 | 61718.10776  47953364.57  1678007.376  2030371.996  725659.8065  54199.83571  265454.1317  296161.147  1574005.458  359413.784  142387.1684  12919.41292  280147885.5  8351.574511  19507.38877  841561.8663  65002.05285  212059.4075  2100.207743  28238.62442  78467.05073  13571.34009  6686048.751  1577.931911  5948245.078  8688.003345  82857.33667  5337230.754  20213384.65  4395118.095  1235092.782  62745.42756  119014.9372  43962.91481  13192.4279  12525.51097  171123.5985  18991.21336  3186082.307  89710.37454  74122.37514  428947.2355  915555.9518  26519703.37  85512.07498  55194.95008  964011.1169  1278855.738  25368.26074  217255.1947  14491428  923097.4084  7582415.76  20190.58818  878591.4235  15137202.14  757124.6229  1456303.966  1955807.089  1023028.903  28141.86578  10557.67691  18023.84497  13106.0118  16673.35772  1380429.437  14548.83822  72195.97521  1240138.572  17330.10464  5975.906728  1235491.666  16756.1243  33041.87719  742727.6213  2405.06161  56533.14465  30443.62102  217090.0018  4384.492704  139662.5968  354.9072713  34769.57411  25351.49409  31900.59116  1130938.075  3061.370823  104511.6976  30140.54258  51286.98321  23985837.14  4311.975383  1637355.409  371056.306  18238.62159  63327.18152  22628.14032  3601619.258  14396.7617  26135.37232  150432.8325  72534.2683  139250.4824  132233.9563  5411155.118  11288898.83  8954309.41  832459.3325  555626.0127  2565726.863  1110.198536  245171.0531  2972813.925  100020.0514  298562.8956  33614.00724  14055.86386  9031010.616  46665366.89  28692.17026  30874.71809  81830.19019  108296.0207  17825.02767  6574.773292  5031627.297  3183180.37  1624411.907  833171.534  200672.8345  877051.8652  75048.04385  1987147.004  1201270.523  1064454.045  1163163.593  15277.39765  7100.066221  1663382.127  23603.14604  60092.04404  105405.5682  40541.87443  890779.5571  30064.5319  1418.909302  1441137.414  78678.05913  443136.3291  2589727.369  23211.48794  269116.3324  278251.4284  918523.8737  9788651.299  39149.70212  11649261.15  28369.55629  18960.07249  124544151.3  2983599.314  48968.59511  216490.0444  60189.29674  7113430.845  4896813.623  7870.672655  147964.2406  214380.5254  71925.93713  162689.8711  142342.4385  3014907.083  652934.4122  344467.4316  19699.18494  105563.4596  5221812.342  1156413.655  50467483.9  10158532.36  20048440.06  42986.17912  468908.3954  16201.92015  481676.2568  222021.2255  251477.0985  50358.68094  3245921.306  15622.64726  177640.0957  257168.8  1654981.851  95767.06587  3418057.704  1820413.969  65134.04687  105325.0344  8076.319415  127603.8487  2006020.76  9041856.805  3727696.491  9581259.421  208968.6549  73243.26762  19661125.33  22810.38358  15206403.21  2082294.315  4277126.481  10682997.14  265238.043  112786528.8  32726.47665  28731.97446  225083.8393  23510786.84  69103.94406  2930318.223  96453.18707  35824.51032  811094.0251  20277717.05  1071407.483  1670614.266  111338.0436  80308.115  3857670.209  154798.6862  692565.8978  180154.8766  40943.64967  5491372.994  21554.41154  1829300.156  210891.3685  5846368.951  249751.1709  13287.50816  452696.1394  4769032.701  3483144.295 | 56889.65495  48378362.62  1462058.77  2538018.338  1066935.581  31353.00415  317894.9145  407892.8414  1629343.555  247914.9587  140838.8301  21577.60446  343249061.3  14245.47723  7981.822818  899246.9216  83635.77382  220067.5627  1561.133366  15037.64754  83413.41946  24349.75778  9091546.645  989.6137979  6450999.975  11282.92758  108542.9403  6817679.068  24654791.95  5217888.283  1436830.795  78124.35556  168239.4808  34552.83214  14514.35725  17566.1955  187499.0833  25708.72731  4471768.554  88629.79684  87877.31814  422715.2208  954536.8509  35159811.19  97688.48792  56328.63558  1003796.329  1457049.738  25633.19909  232440.0183  15296834.43  846636.0143  7956691.189  19625.97054  1145269.298  15293525.29  660621.3576  1766746.363  2457501.907  1264038.555  36814.8916  10703.2766  24918.18541  14686.97735  23291.87878  1470595.099  18293.33195  86591.69524  1204380.216  20970.81263  8249.132565  1414214.725  9141.766199  28090.95564  844432.8683  808.0039443  75454.5135  29592.72204  283761.3403  3232.466364  160447.057  658.8870408  45556.72874  37288.11273  33223.2939  858562.0269  1888.436614  136175.651  30134.96682  50386.01208  34495082.35  3818.921359  2130061.167  389785.4442  27347.78156  119372.4189  18211.94987  3442131.376  16237.46781  13721.64496  172524.6327  82460.78399  124954.6869  105787.9994  3914856.392  10871232.59  10231284.46  840426.6774  689069.139  3017241.902  253.2552916  218254.6231  2504178.706  175433.2809  275881.0608  30878.45169  20825.88804  7428174.528  41580376.02  32256.03955  17859.82488  97432.93061  87395.39471  22410.22436  9182.384632  4763433.348  3302482.523  2003787.825  782905.805  156952.4438  1288681.12  65570.61403  2151986.492  1326024.886  992044.7294  1511671.85  32095.1834  11404.24772  1501487.217  29755.4149  66275.69723  62719.31278  64516.21482  1005441.523  18819.79004  4672.064644  1555760.413  93644.26893  815601.4769  4655434.328  16925.23725  283047.6982  314212.5098  769746.2253  9502536.146  61477.70844  15235903.29  21450.41815  20261.43538  166488753.3  3257307.782  45567.33931  241021.7138  77282.04861  4628853.044  9764643.281  6735.170032  143136.6068  201549.3392  76337.57821  157703.1472  141685.1821  2825020.62  618361.1298  286345.2449  15596.19995  119402.967  6254068.448  1781085.761  58124458.89  13800965.14  24686590.27  73308.43123  424948.3886  15328.30599  954509.4787  202673.4153  255004.5971  45063.42358  1880335.917  49303.21442  227503.9615  323926.3084  2333826.987  69937.70011  3100708.653  1867354.155  55906.46324  140883.2352  6441.45642  156121.2005  2933281.111  13139698.85  6552526.716  15272916.87  199086.4728  65887.92616  21269398.18  26998.52679  17045711.62  1089881.019  3667400.899  9241455.968  314285.8378  124192213  48613.64651  28507.95684  197279.2318  23752322.17  108723.1829  3742119.191  211305.1252  46256.07923  1219427.013  31162121.26  1134302.433  1323007.483  114429.733  54727.47774  3939626.847  176237.6734  701738.7359  123028.7473  38507.55396  6715522.37  35931.7227  1901962.829  155343.6883  6696463.612  213499.0526  9254.610203  846463.1322  2578648.604  3774724.818 | 69116.5109  50179698.24  3123013.767  2739995.775  665136.1026  91010.92834  345983.7629  407603.7496  1765230.269  697551.454  283979.7329  29148.42961  357383258.4  14244.23429  23227.76815  1207012.031  96352.3663  343311.5542  3935.528803  21425.29156  117732.6954  38964.6702  9107152.969  2603.679905  8289711.319  9765.755797  121388.2344  7095061.833  24846926.03  6969722.187  1482460.736  104169.0919  185793.914  66710.70169  30484.18838  34497.00313  181432.8896  29209.53184  4339618.723  111154.7106  103339.316  552432.5906  1364134.151  38396535.37  83250.07077  86798.58159  1135699.172  1390167.089  31460.82671  223444.1964  17913218.21  1152661.568  9389648.303  29034.97015  1330189.187  16372567.23  907122.44  1852763.621  3145108.052  1443901.7  62025.72224  16388.62122  32828.33975  14979.24936  23313.99659  1588357.997  29465.07441  92710.49296  1573294.699  36650.59033  5105.544525  1465175.053  15912.95633  23471.81548  914520.6656  1562.532675  93874.17298  47666.60713  331943.0365  13816.87509  142791.3984  1353.185144  53143.8253  41510.70433  48140.07129  1156170.901  734.1832653  180376.7327  38530.03519  50861.95601  29029466.84  9544.557446  2430970.037  489620.8802  21194.73676  132905.5516  55644.62924  4323782.234  23590.5573  25674.57988  120655.8768  78606.59936  201590.886  113461.2934  5949063.127  14767782.74  14629154.52  1125813.468  1047462.489  3646322.033  3490.027621  331342.0874  3515426.78  166045.0797  324988.8821  54013.56377  15583.36304  9672968.157  55652110.41  47219.38677  29241.54758  88045.84015  135688.1861  27937.02603  16067.59755  5402605.42  4188452.148  2170445.722  944155.345  222977.6706  1305500.133  93318.14194  2194622.083  1439773.17  1237376.643  1253411.827  11686.53503  9643.863178  3197755.071  25925.47644  65951.81062  93992.4107  76120.30713  1289952.43  30802.1498  2740.404464  2164240.331  172787.6449  565772.0137  4094900.74  13833.99224  370778.269  388778.0905  974132.4259  10164248.51  56679.09508  19565050.36  34279.83796  30867.49126  144430454.3  3312692.037  65622.75467  290787.6394  58244.75539  7917439.358  7724287.978  8576.981719  158067.5992  322886.5295  102218.1984  213278.9918  167576.6971  3656452.004  1179833.192  399875.0819  22712.35439  146827.512  7390143.326  1695665.981  57480779.33  14415460.78  30002634.87  78452.32737  536676.6306  33405.3442  805188.9311  277836.6894  374544.9352  42493.13222  3616128.963  14965.97324  213393.8874  396295.7269  2508817.401  151138.2645  4639783.011  2289325.528  97598.65018  130982.183  29845.37675  130058.4846  2664582.907  13309633.79  4195015.695  12051791.04  258161.2262  87862.33419  24890541.82  56545.53451  18748023.99  1795633.771  4232333.284  11512168.13  576618.5276  120772342  61884.6525  48480.15312  340197.8553  29107746.3  117039.7332  5090128.037  172218.4554  60273.79089  1139956.801  17373405.82  1213964.044  1493553.632  173333.8674  47554.45988  5793606.224  153345.0211  973135.3684  189731.0961  33155.41533  7075203.354  56969.61979  2438236.143  272964.4691  8025645.284  208006.4222  17488.50674  844405.448  4829016.762  4902820.829 | 42305.41404  59225631.83  1620297.932  2232673.481  859147.7161  48325.18298  351941.157  322571.1232  1440675.025  336338.1383  159564.5668  18273.55407  284686368.2  15456.19377  15847.7609  874780.5473  83240.3346  257799.3713  2595.6051  8585.584296  114106.0627  18251.81743  8487213.165  1034.543976  6632012.35  15980.18139  101232.7369  6991310.58  22776623.89  5352605.622  1427375.285  63102.92985  137697.3233  57659.16905  18519.54596  21615.6032  196226.4641  27036.80672  3583368.637  109470.0329  73419.61655  516408.7404  1088151.19  33612686.76  98970.21058  61740.42328  1455422.394  1265078.075  14512.1288  254585.1236  14071542.5  1158137.58  8722206.93  25010.69032  830236.8923  15207158.78  1032410.953  1717484.982  2384235.863  1432339.685  26285.0124  12888.70549  27506.91476  15428.59695  28300.21091  1582653.963  21002.23457  91276.96707  1333761.666  22509.24685  13719.99753  1690707.682  12854.61338  17471.68029  982425.5557  1377.451856  60768.37499  51834.8305  211968.2987  6027.114062  137770.5284  1157.685748  41940.28853  52465.27283  58861.3125  1486595.319  2570.532673  103641.9983  40241.01392  71005.39623  32254417.57  3870.447768  1731843.312  425363.0024  23904.04925  97596.44085  36028.57932  4577726.876  17293.88494  22285.63492  168157.8024  62681.9027  129867.3608  182510.8395  4940443.519  15629793.12  10311661.73  1313958.734  699135.1602  2542547.156  329.2936269  325532.627  3658637.342  153337.227  466346.9161  48913.46061  14552.4422  11297226.32  58660122.95  23843.0459  39745.14588  113101.0333  134380.8157  23653.80903  11595.11699  8078845.271  3477536.914  2047870.3  1117117.097  321588.8555  1000553.496  96301.02403  2408336.195  1261174.543  1246933.264  1403348.477  9991.343715  12541.15487  1908405.548  21070.28975  83359.94251  101056.0482  58022.77607  1183824.806  31266.8642  2013.527598  1634010.848  116261.6752  479535.5072  3544754.344  23565.88219  273907.5101  402705.6649  1159853.94  14177738.33  66326.19265  15765331.5  40310.84342  20852.87139  157248774.5  3396365.382  88160.91788  292005.0584  43928.57081  7861064.36  5947912.739  12655.61177  219705.0998  209269.2305  92832.82758  148543.3383  223516.5843  3070957.433  716744.6367  502576.9948  31732.10041  97232.78001  8084253.381  1628958.147  69174420.76  12310761.76  31719618.02  53446.43723  747039.6322  18726.86994  574146.4833  253693.5103  322945.7421  63091.04562  3186471.007  14995.84451  212527.116  325770.3872  2704957.394  164702.1178  4235856.978  2189858.381  83429.36568  150224.6771  9607.510002  150797.1324  2636669.768  9630832.532  5078211.441  10858323.24  261055.1629  65577.65155  24137908.67  44087.70998  18892900.43  3032992.015  4733869.098  14774227.18  302816.1336  122184790.6  42054.84936  41596.59605  308341.1444  24951450.93  84419.57866  4977307.186  142952.8011  46537.27265  998123.3259  18855068.78  1181977.193  1890578.831  156018.9368  63143.93016  4429328.31  118860.6781  754963.952  282870.0967  65070.49378  6423181.447  30063.78363  1561566.447  229321.956  7027962.49  329427.9661  16596.9398  555649.5369  6359367.872  5130487.004 | 2.85E-08  1.74E-07  2.84E-07  8.44E-07  1.43E-06  2.35E-06  3.84E-06  4.13E-06  4.16E-06  4.41E-06  5.59E-06  6.18E-06  8.18E-06  9.39E-06  1.02E-05  1.05E-05  1.48E-05  1.85E-05  1.98E-05  2.74E-05  3.11E-05  3.33E-05  3.66E-05  3.68E-05  5.39E-05  5.46E-05  5.46E-05  5.51E-05  5.56E-05  6.00E-05  6.68E-05  6.91E-05  7.01E-05  7.23E-05  7.40E-05  8.73E-05  0.00010045  0.00011  0.00011926  0.00012837  0.00013512  0.00015184  0.00015788  0.00016661  0.0001798  0.00021682  0.00022125  0.00022919  0.00024305  0.00025076  0.00028056  0.00028512  0.00030227  0.00030816  0.00031713  0.00032984  0.00033746  0.00034508  0.00039648  0.00041921  0.00042264  0.00047213  0.00050261  0.00055836  0.0005814  0.00064434  0.00064688  0.00075897  0.00075953  0.00078088  0.00078452  0.00078779  0.00085265  0.00089782  0.00091978  0.00092385  0.0010148  0.001026  0.0011553  0.0011656  0.0011702  0.0012388  0.0013241  0.0013464  0.0014259  0.0017344  0.0017358  0.0017429  0.0017435  0.0018957  0.0019298  0.0019738  0.002054  0.0022511  0.0023241  0.0023452  0.0023529  0.0023865  0.0024383  0.0024758  0.0024851  0.0024982  0.0025723  0.0028274  0.0028697  0.0030103  0.0031474  0.0033746  0.0035502  0.003613  0.003749  0.0038355  0.0038451  0.0039729  0.0040749  0.0041411  0.0042262  0.0042596  0.0043722  0.0043735  0.0043889  0.0047091  0.0047758  0.0049858  0.0051568  0.0052057  0.0053864  0.0054246  0.0055891  0.0055902  0.0056193  0.0056253  0.0058487  0.005902  0.0059229  0.0060308  0.0061236  0.0062018  0.0066551  0.0068435  0.0069721  0.0072745  0.007627  0.0076831  0.0080079  0.0081493  0.0087367  0.0087465  0.0088153  0.00884  0.0088869  0.0092635  0.0093733  0.009422  0.0094695  0.0096597  0.0099516  0.0099648  0.010233  0.010345  0.010567  0.010769  0.011249  0.011657  0.011774  0.011879  0.011946  0.012043  0.012133  0.012606  0.013148  0.013343  0.013371  0.01343  0.013531  0.013681  0.014076  0.014238  0.014702  0.014828  0.015322  0.01538  0.015756  0.01588  0.015899  0.016211  0.016478  0.016663  0.017017  0.017299  0.017384  0.017916  0.018056  0.018092  0.018435  0.018478  0.018755  0.019228  0.019264  0.019551  0.019777  0.020087  0.020185  0.02183  0.022488  0.023148  0.023182  0.023405  0.023691  0.023962  0.024289  0.024908  0.024953  0.027522  0.028277  0.028299  0.028661  0.029388  0.030993  0.03133  0.031489  0.032108  0.03348  0.033979  0.034874  0.036262  0.036505  0.036994  0.037481  0.037766  0.037899  0.038618  0.039009  0.039225  0.039595  0.03965  0.041814  0.043139  0.04413  0.046835  0.047475  0.048331  0.048473  0.049573 | 1.588622246  1.586506575  1.58306792  1.577207705  1.576138608  1.583298  1.567168623  1.573313581  1.571642549  1.574521376  1.570898283  1.5703904  1.567720073  1.557725005  1.573971332  1.564809155  1.555454852  1.554391102  1.564411901  1.558449167  1.55741802  1.555000087  1.540121253  1.550097638  1.551728776  1.539963094  1.542099949  1.547547197  1.539813195  1.549103645  1.539228988  1.542630777  1.533580344  1.552184411  1.552185566  1.545223975  1.538062162  1.537563855  1.533970442  1.534924593  1.527914711  1.534105667  1.53437042  1.527435116  1.537664161  1.526582756  1.520232918  1.524684878  1.511540652  1.526600651  1.523988129  1.510311988  1.508567339  1.51380946  1.516813633  1.510290904  1.499500912  1.496704327  1.509527211  1.511709536  1.507783664  1.511142052  1.496757434  1.50834035  1.49197303  1.488214096  1.48035201  1.487997964  1.494753841  1.472701296  1.4598073  1.464608782  1.497144084  1.480333881  1.484042307  1.482540444  1.465702379  1.489454793  1.472814761  1.479105406  1.471038961  1.472546128  1.471519138  1.451005059  1.457728822  1.455603622  1.446198903  1.454235665  1.454248723  1.459742595  1.46834247  1.465264802  1.445115024  1.441113775  1.428841582  1.445455996  1.451935668  1.408713528  1.431669946  1.401827558  1.451741625  1.435974306  1.440137505  1.420664724  1.400337918  1.416291521  1.425118943  1.410591871  1.416756359  1.422010092  1.414798679  1.384696187  1.379263905  1.426968712  1.40157635  1.372511959  1.426589062  1.390674241  1.396001452  1.406142233  1.401814992  1.382026814  1.387648397  1.390263372  1.390356204  1.376270048  1.387939813  1.374118732  1.372718765  1.387604661  1.364757255  1.351558281  1.361566725  1.392473219  1.339742944  1.346740976  1.351906067  1.386266999  1.379526177  1.363621139  1.371398257  1.326849104  1.364036409  1.354407729  1.307289952  1.345666741  1.353499842  1.350647645  1.346804617  1.365635626  1.34716149  1.344408545  1.330133683  1.332508625  1.339966794  1.326570615  1.322676668  1.329049084  1.293506034  1.350442795  1.31438899  1.327515072  1.332261138  1.325181217  1.27843812  1.346340999  1.298110177  1.310552877  1.341176718  1.290099344  1.322167832  1.315719257  1.310893696  1.326073061  1.299570764  1.292541166  1.300293583  1.309232244  1.300388616  1.303666127  1.253962319  1.300382504  1.291044365  1.282124864  1.293766808  1.312648747  1.294994593  1.296205139  1.288940502  1.239515024  1.244600328  1.295838528  1.281669958  1.29257947  1.249981789  1.24516574  1.229978986  1.250505342  1.274120746  1.291217336  1.211134572  1.22590195  1.25327687  1.286793607  1.236180625  1.212525321  1.254528954  1.228848577  1.234578123  1.248917093  1.217802585  1.223551873  1.229443196  1.207161782  1.201543563  1.222954756  1.18487957  1.165344864  1.216523862  1.197677506  1.199905496  1.231527187  1.207604062  1.209299422  1.178424838  1.143620283  1.167153934  1.179411107  1.15922774  1.17895034  1.164164411  1.185368388  1.159376594  1.184002634  1.182646986  1.149948469  1.181338274  1.162776912  1.139016263  1.163140185  1.132933556  1.158207306  1.094315426  1.139578466 |
