# Supplementary figures and images for: Effects of Sevoflurane Anesthesia on Cerebral Lipid Metabolism in the Aged Brain of Marmosets and Mice
Source: Front Mol Neurosci. 2022 Jul 6;15:915570. doi: 10.3389/fnmol.2022.915570 (PMC9298509; doi:10.3389/fnmol.2022.915570)

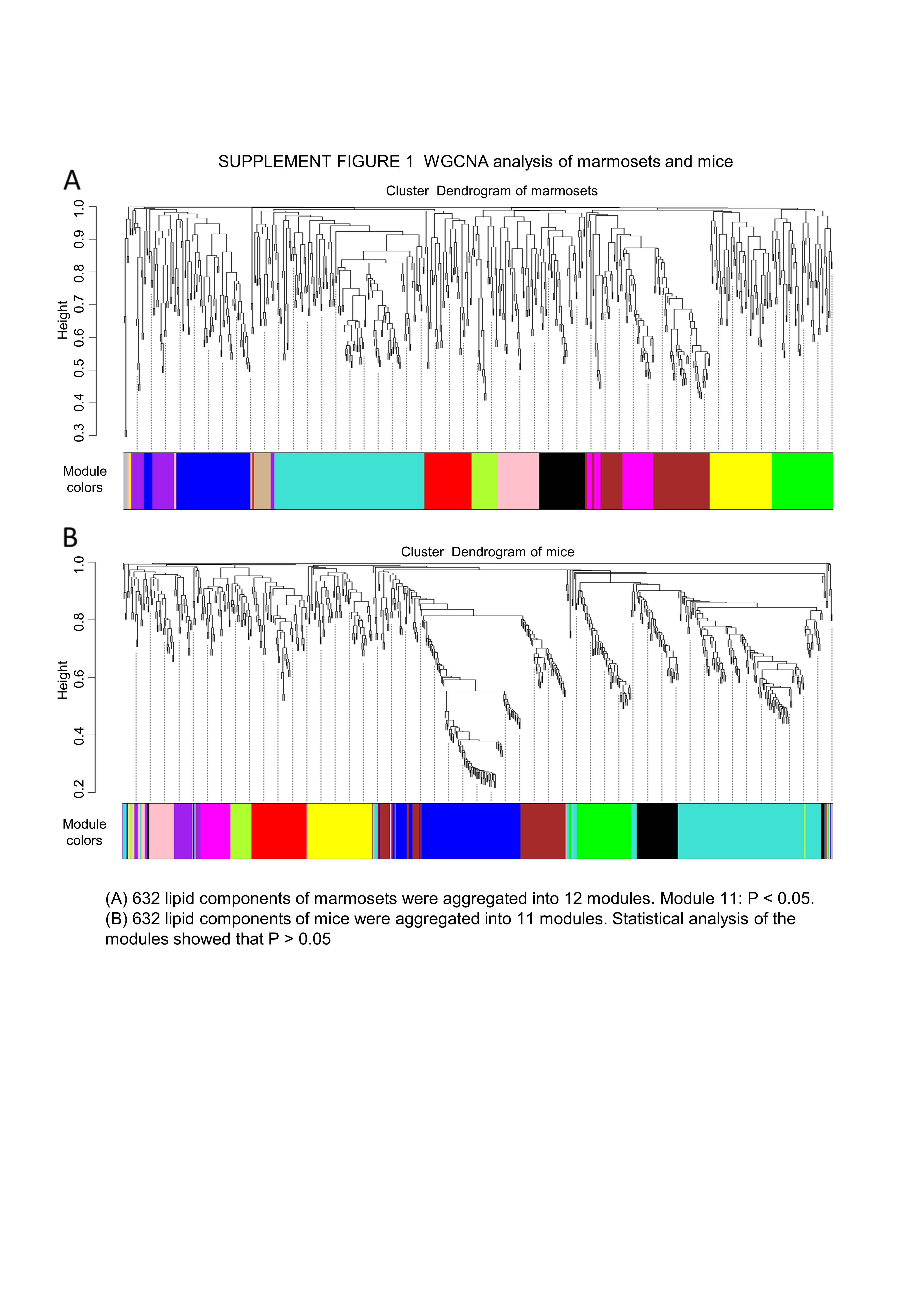

Supplement: Supplementary Figure 1 — WGCNA analysis of marmosets and mice. (A) A total of 632 lipid components of marmosets were aggregated into 12 modules. Module 11: P < 0.05. (B) A total of 632 lipid components of mice were aggregated into 11 modules. Statistical analysis of the modules showed that P > 0.05. [file Image_1.jpg]
